# The Potential Health Benefits of Gallic Acid: Therapeutic and Food Applications

**DOI:** 10.3390/antiox13081001

**Published:** 2024-08-18

**Authors:** Milad Hadidi, Rafael Liñán-Atero, Mohammad Tarahi, Marios C. Christodoulou, Fatemeh Aghababaei

**Affiliations:** 1 Institute of Physiological Chemistry, Faculty of Chemistry, University of Vienna, 1090 Vienna, Austria; 2 Department of Organic Chemistry, Faculty of Chemical Sciences and Technologies, University of Castilla-La Mancha, 13071 Ciudad Real, Spain; rafael.linan@uclm.es; 3 Department of Food Science and Technology, School of Agriculture, Shiraz University, Shiraz 7144165186, Iran; tarahimohammad@yahoo.com; 4 Department of Chemistry, University of Cyprus, Nicosia 1678, Cyprus; mchris39@ucy.ac.cy; 5 Aora Health, Scientific Park of Madrid, Faraday 7, 28049 Madrid, Spain; aghababaei.afi@gmail.com

**Keywords:** antioxidant, anticancer activity, phenolic compounds, pharmaceutical applications, human health

## Abstract

Gallic acid (GA), a phenolic acid found in fruits and vegetables, has been consumed by humans for centuries. Its extensive health benefits, such as antimicrobial, antioxidant, anticancer, anti-inflammatory, and antiviral properties, have been well-documented. GA’s potent antioxidant capabilities enable it to neutralize free radicals, reduce oxidative stress, and protect cells from damage. Additionally, GA exerts anti-inflammatory effects by inhibiting inflammatory cytokines and enzymes, making it a potential therapeutic agent for inflammatory diseases. It also demonstrates anticancer properties by inhibiting cancer cell growth and promoting apoptosis. Furthermore, GA offers cardiovascular benefits, such as lowering blood pressure, decreasing cholesterol, and enhancing endothelial function, which may aid in the prevention and management of cardiovascular diseases. This review covers the chemical structure, sources, identification and quantification methods, and biological and therapeutic properties of GA, along with its applications in food. As research progresses, the future for GA appears promising, with potential uses in functional foods, pharmaceuticals, and nutraceuticals aimed at improving overall health and preventing disease. However, ongoing research and innovation are necessary to fully understand its functional benefits, address current challenges, and establish GA as a mainstay in therapeutic and nutritional interventions.

## 1. Introduction

Concerns related to human health have reached an unprecedented level due to the increase in chronic and degenerative diseases, including cancer, cardiovascular diseases, and antimicrobial-resistant infections. These health problems have led the scientific and medical community to seek out solutions for the prevention and treatment of these conditions [1]. In addition, the trend towards the use of natural remedies has intensified, as people seek to reduce their exposure to synthetic chemicals, opting for healthier and more environmentally friendly alternatives [2]. In this context, phenolic compounds have been promising candidates for years, due to their multiple biological and therapeutic properties [3,4]. Phenolic compounds, present in a wide variety of plants and foods, have been extensively studied for their ability to act as antioxidants [5], anti-inflammatories [6], and antimicrobials [7]. These compounds play a critical role in neutralizing free radicals, modulating inflammatory pathways, and inhibiting the growth of pathogenic micro-organisms [8]. There are currently about 8000 known phenolic structures, ranging from basic phenolic acids to highly polymerized compounds [9]. Among them, gallic acid stands out for its potent biological activities and therapeutic versatility.

Gallic acid (GA) is a phenolic compound abundantly found in various plants such as tea leaves [10], oak bark [11], or the galls of some trees and shrubs [12,13], as well as in some foods such as walnuts, apples, strawberries, pineapples, bananas, blackberry, lemons, or grapes [14,15,16,17]. It is generally obtained in both free and conjugated forms, either as an ester or as derivatives of catechin (catechin gallates). The most common ester derivatives of GA are the alkyl esters, e.g., methyl, propyl, octyl, and dodecyl gallate [18]. The concentration of GA or its derivatives found in different plant tissues varies depending on biotic and abiotic stress factors like UV radiation, microbial infections, and other environmental stresses. In addition, the extraction method used also has a significant influence on the amount of GA obtained. Thus, both conventional extraction methods such as soaking [19] and Soxhlet extraction [20] and novel methods such as microwave extraction (MAE) [21], ultrasonic extraction (UAE) [22], and ionic liquid extraction (IL) [23] have been used, resulting in a different GA content based on the extraction technique and part of the plant used.

The pharmaceutical industry frequently uses GA due to its remarkable anti-inflammatory and antioxidant properties, which have been demonstrated in numerous in vivo and in vitro research studies including humans, animals, and cell cultures [24,25,26,27,28,29]. In traditional medicine, it has been used to treat diseases such as diarrhea, dysentery, and internal bleeding, as well as to reduce inflammation and other gastrointestinal diseases [26,27]. Similarly, it has been used in the treatment of conditions related to ageing and cell damage because of its ability to scavenge free radicals [28]. Due to its antiseptic properties, it has also been useful for cleaning, disinfecting, and healing wounds, thereby reducing infections [29]. To treat gastrointestinal problems, GA has been commonly found in various herbal preparations, such as infusions and teas made from leaves and fruits rich in GA (e.g., berries), while for the treatment of wounds and skin infections, topical ointments, powders, and pastes enriched with this phenolic compound have been used [30]. The biological activity of GA is not limited to the above effects but has also been shown to exert antimicrobial [31,32,33], antimelanogenic [34,35], antiviral [36,37], anti-allergic [38,39], and neuroprotective effects [40,41]. Due to its bioactive and functional properties, it has been widely applied in various areas of the chemical industry [42]. For example, its ability to form complexes with iron has been used in the measurement of gallocatechin content in beverages like tea [43]. In addition, GA has been shown to be of great importance in the production of writing inks, due to the blue complex that originates when GA binds to iron [44]. In chemical research, GA is used as a standard in analyses to measure the phenolic content in plant extracts by the Folin–Ciocalteau assay, expressing the results in gallic acid equivalents (GAEs) [45]. 

This review explores the chemical structure and natural sources of GA, along with offering a detailed examination of multiple identification and quantification techniques. In addition, this review delves into the biological and therapeutic properties of GA, highlighting its antibacterial, antioxidant, and anticancer potential. In addition, various food applications of GA are also discussed, particularly in active food packaging and its use in functional food formulations.

## 2. Chemical Structure and Various Sources

**Figure 1:**
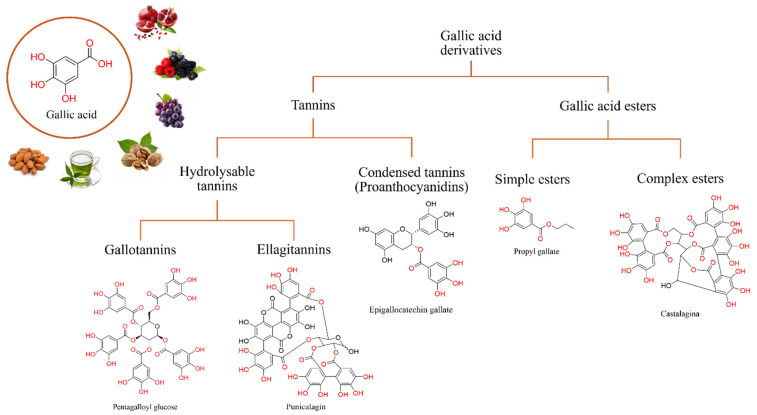
General classification and main natural sources of GA and its derivatives.

GA, or 3,4,5-trihydroxybenzoic acid, is a yellowish-white crystalline compound discovered by C. Wilhelm Scheele in 1787 [46]. Its chemical formula is C_7_H_6_O_5_, and it has a molecular weight of 170.12 g/mol. It is formed by a tri-hydroxylated phenolic structure at positions 3, 4, and 5 of the benzene rings and a carboxyl group, and theoretical studies on GA conformation suggest the preference for a planar geometry [47]. GA is widely distributed in nature, where it is found in the form of free acid or esters (gallates) or in conjugated form with tannins [48]. Figure 1 shows the structure of GA along with its main derivatives, as well as the main natural sources from which they are extracted.

GA is usually obtained from various parts of plants, such as bark, seeds, and flowers, as well as food such as walnuts, almonds, red wine, green and black tea, grapes, or berries [49]. The extraction of GA from different agricultural sources has been traditionally carried out by different researchers, using both conventional and modern extraction techniques [50]. For instance, Amirah et al. [51] compared the efficiency process of different extraction methods to obtain the highest amount of GA from *Jatropha curcas* stem bark. The extraction methods used were maceration, UAE, and MAE, and each technique was evaluated in terms of GA yield, extraction time, solvent consumption, and energy efficiency. The main results showed that UAE and MAE provided significantly higher yields of GA (0.40 mg/mL and 0.54 mg/mL, respectively) compared to traditional maceration (0.22 mg/mL). In addition, MAE extraction was found to be significantly more efficient than maceration, achieving higher yields in a shorter extraction time (2 min vs. 6 h). Generally, traditional methods involve high temperatures, complex experimental requirements, longer extraction times, and the use of large amounts of organic solvents. These factors can lead to the degradation of heat-sensitive compounds, increased energy consumption, and greater environmental impact [52]. To address the limitations of conventional extraction methods, several innovative techniques have been developed, including microwave-assisted extraction (MAE), ultrasound-assisted extraction (UAE), supercritical fluid extraction (SFE) [53], ionic liquid extraction (IL) [54], pulsed electric field extraction (PEF) [55], and enzyme-assisted extraction (EAE) [50,56]. In both conventional and novel methods, it is crucial to consider the location of the target compound within the plant tissue. Additionally, the extraction yield is influenced by factors such as the polarity of the compounds, extraction time and temperature, sample-to-solvent ratio, and physicochemical properties of the samples [50]. Therefore, novel techniques do not always achieve higher extraction yields than conventional methods, underscoring the importance of carefully evaluating all these factors. In this regard, Khodaie and Ghoreishi [57] compared the amount of GA obtained from brown sumac seeds using two extraction methods, a traditional method—Soxhlet extraction—and a more novel method—supercritical carbon dioxide extraction (SFE-CO_2_)—observing that the amount of GA obtained by the Soxhlet method was approximately two times higher than that obtained by SFE-CO_2_ (15.29 mg GA/g sample versus 30.25 mg GA/g sample). Despite these results, the SFE-CO_2_ extraction method was much more efficient than the Soxhlet method due to the shorter extraction time (2 h vs. 8 h), the smaller amount of solvent used (210 mL vs. 250 mL), and the low temperature used in the extraction (35 °C vs. 60 °C). In addition, SFE-CO_2_ extraction allowed the obtaining of a high-purity extract, without the need to apply a purification step, as it would have to be done for the extracts obtained by Soxhlet extraction [57]. Therefore, for each extraction process of a compound, it will be necessary to find a balance that allows an acceptable amount of the compound to be obtained, using processes and reagents that are as sustainable as possible in terms of energy efficiency and environmental friendliness.

**Figure 2:**
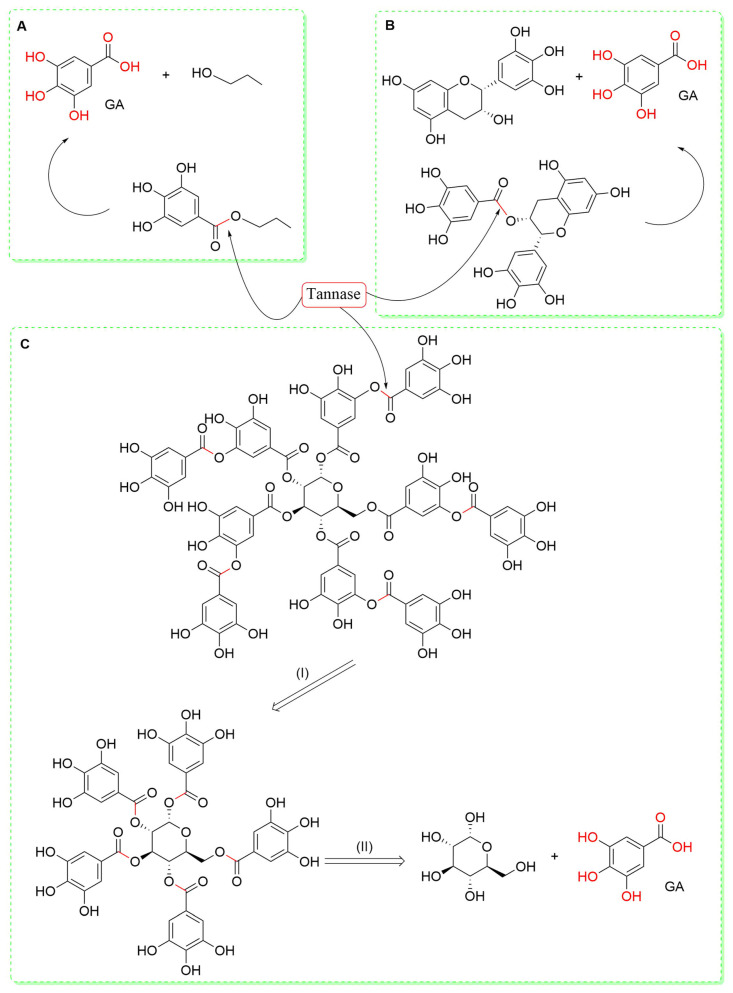
Production of GA from some of its derivatives by tannase-catalyzed reactions: propyl gallate (**A**), epigallocatechin gallate (**B**), and tannic acid (**C**).

In addition to the extraction methods mentioned above, GA can be obtained by the chemical acid hydrolysis of some GA derivates, such as tannins or GA esters, as well as by the transformation of substrates by microorganisms [58]. Tannins are a large group of polyphenolic compounds, subdivided into hydrolysable tannins, which comprise polymers of ellagic acid and GA (ellagitannins and gallotannins, respectively), and condensed tannins or proanthocyanidins, formed by the polymerization of flavan-3-ols such as catechin and epicatechin (see Figure 1) [30]. Both tannins groups are found in abundance in all parts of plants (roots, flowers, fruits, leaves, peels, skins, and stems), and because of their biological activities, different uses or applications have been described for these compounds. The conventional extraction procedures are sometimes unfavorable due to their low purity, low yield, high cost, and the release of hazardous effluents into the environment. Alternatively, the enzymatic biodegradation of tannins and other GA derivatives is another method to obtain GA [59]. The degrading of these compounds is carried out by tannin acyl hydrolase (EC 3.1.1.20), commonly called tannase, which catalyzes the hydrolysis of the ester bonds present in GA derivatives (Figure 2).

This enzyme can be produced by fungi, yeasts, and bacteria, and depending on the organism that produces it, tannase may display different activities [60]. In this regard, fungi effectively degrade hydrolysable and complex tannins such as (−)-epicatechin gallate and epigallocatechin gallate but not condensed tannins. Conversely, bacteria only break down hydrolysable tannins, while yeasts are only able to hydrolyze gallotannins [61]. Industrially, most tannase-producing microorganisms are fungi, but their use has two drawbacks: the slow degradation of tannins and the difficulty of manipulating them genetically. For this reason, a large number of bacteria capable of producing tannase have been studied, such as *Bacillus subtilis* and *Lactiplantibacillus plantarum* [62], *Pseudoduganella albidiflava* [63], and *Clostridium butyricum* [64], among many others [58].

## 3. Identification and Quantification Techniques

Analytical studies are essential to obtain qualitative and quantitative data on the presence of specific compounds in foods and plant matrices. In this regard, several instrumental techniques, such as chromatography, capillary electrophoresis, and spectroscopy are widely used for the determination and quantification of compounds. Specifically, numerous studies have used different techniques to isolate and analyze GA [65,66]. Representative examples of methods for the detection and quantification of GA in different agri-food matrices are listed in Table 1. The table also contains a summary of the sample preparation steps and the GA concentration found in each sample.

### 3.1. Chromatography Techniques

#### 3.1.1. High-Performance Liquid Chromatography (HPLC)

The most used techniques for identifying and measuring GA in various matrices are chromatographic methods. Among these methods, HPLC is the most extensively used technique used for the measurement of GA and other phenolics compounds. This technique is well known to be highly sensitive, allowing the detection and quantification of compounds at very low concentrations. This is critical for the analysis of analytes such as GA, which can be present in complex matrices at very low concentrations (µg/g) [71,72,75]. Moreover, the sensitivity of HPLC is another aspect that makes the technique stand out. This is due to the coupling of HPLC to advanced detectors like the diode array detector (DAD) [68,69] and the mass detector (MS) [73,74], which can identify and quantify even trace amounts of the compound. The advantages of HPLC-MS are partly due to the combination of the physical separation provided by liquid chromatography (LC) and the mass analytical capabilities of mass spectrometry. HPLC also stands out as a very versatile technique due to the wide variety of columns available. Among them, reverse-phase chromatography (RP) is the most popular choice for determining GA and other polyphenols in agri-food matrices. In this modality, the stationary phase consists mainly of saturated aliphatic groups chemically bonded to silica or polymeric particles, with the octadecyl group (C18) being the most commonly used. The analysis of GA is commonly performed by RP-HPLC, due to its chemical properties and the characteristics of the chromatographic technique itself. GA is relatively polar, with hydroxyl groups that confer a certain solubility in aqueous and organic polar solvents. These properties make GA particularly suitable for analysis by RP-HPLC, which uses moderately polar aqueous or organic mobile phases and non-polar stationary phases. In addition, the sensitivity of RP-HPLC is high for compounds that absorb in UV (λ_GA, max_ = 270 nm), allowing accurate and reliable quantification [88]. Other LC separation modes, such as normal phase (NP) and hydrophilic interaction liquid chromatography (HILIC), may provide complementary separations to RP chromatography [89]. However, neither is a suitable choice, since NP-LC is more suitable for apolar or low-polarity compounds that interact strongly with polar stationary phases and HILIC is ideal for strongly polar and hydrophilic compounds.

Based on Table 1, the columns chosen for GA determination consist of a C18 stationary phase with a column length ranging from 100 to 250 mm, an internal diameter ranging from 2.1 to 4.6 mm, the most common being 4.6 mm, and a particle size ranging from 2.1 to 5 µm. Regarding the mobile phase, hydro-organic mixtures with MeOH or ACN as the organic component, with small amounts of an organic acid, are the most widely used mobile phases in RP-HPLC. The composition of the mobile phase can be constant (isocratic elution) or variable (gradient elution), although gradient elution systems are more commonly used than their isocratic counterparts. In this regard, it is common to use linear gradients with an aqueous phase (called A) and an organic phase (called B), both possibly acidified with small amounts of organic acids such as formic acid (FA) or acetic acid (AA). Acidifying the mobile phase ensures that the hydroxyl groups of the GA remain in their non-ionized form, which improves retention and resolution on RP columns [90]. Analysis times also vary widely, ranging from 10 to 60 min, and the flow rate used usually ranges from 0.3 to 1.5 mL/minute (most commonly, 1 mL/minute). Although in many cases they are not stated, typical sample injection volumes range from 10 to 20 µL, and the column temperature is between 20 and 60 °C. All these parameters, together with the plant/food matrix, the pre-treatment of the sample, and the extraction technique used, influence the final concentration and amount of GA obtained.

#### 3.1.2. Gas Chromatography (GC)

GC is an analytical technique characterized by its reliability, sensitivity, and identification of complex sample mixtures [91]. In particular, GC coupled to MS (GC-MS) is a fast and accurate method that is widely applied for the detection and quantification of a wide variety of compounds. However, not all compounds can be analyzed by GC, since the use of this method requires that the substance has a good volatility and does not boil at temperatures above 300 °C [92]. For that reason, the sample processing for GC-MS analysis includes solvent extraction of the sample, concentration to dryness, and consecutive derivatization, which is an essential process that reduces the polarity of the analyte of interest, while increasing its volatility and thermal stability. The derivatization of the analyte is usually performed in a two-step procedure: (1) the selection and preparation of the derivatization reagent and (2) the derivatization reaction. Common derivatization reagents include silylation reagents (such as N,O-bis(trimethylsilyl)trifluoroacetamide, BSTFA, or N-methyl-N-(trimethylsilyl)trifluoroacetamide, MSTFA), acylation reagents (such as acetic anhydride), and alkylation reagents (such as diazomethane) [91]. For instance, de Souza Dias et al. [76] analyzed several phenolic compounds (including GA) present in wines by GC-MS. GA has a high boiling point, in the range of approximately 300 °C to 320 °C. This high boiling point indicates that a very high temperature is required to vaporize the compound, which may cause thermal decomposition before reaching the gas phase necessary for GC-MS analysis. Therefore, the authors of the study derivatized GA using BSTFA, which increases the volatility and thermal stability of GA, thus making it more suitable for GC-MS analysis. The analysis of phenolic acids by GC-MS (specifically GA) almost always involves a derivatization process, so it is more common to use other techniques for the analysis of this class of compounds. Methods such as HPLC-MS or HPLC with UV–Vis or DAD detectors are more suitable and recommended for GA analysis [93].

#### 3.1.3. High-Performance Thin-Layer Chromatography (HPTLC)

A popular method for fingerprinting that makes use of tiny volumes of materials and solvents is thin-layer chromatography (TLC), which allows rapid and simultaneous analysis [94]. These days, modern TLC is often used and useful as HPTLC, which is limited to precoated layers, instrumented, and mostly used for quantification. HPTLC is known for its uniformity, purity profile, precision and accuracy of results. Moreover, together with TLC, it is characterized as being the fastest chromatographic method, since the chromatography of the samples is performed in parallel [95]. In TLC/HPTLC, the plate can be developed chromatographically in either a vertical or horizontal orientation. The development can also be carried out in one dimension, with a single mobile phase (isocratic), or repeatedly in the same direction with varying mobile phases (gradient). It can also be carried out by a linear, circular, or anti-circular movement of the mobile phase [95]. Numerous high-performance (HP)TLC methods for the analysis of phenolic acids are available in the literature. For example, a study by Chaphalkar et al. [96] analyzed total phenols, flavonoids, and tannins by standard assays, while HPTLC was used to quantify the presence of GA. In the study, aluminum HPTLC plates precoated with silica gel F60_254_ (10 × 10 cm) of 200 μm thickness were used as the stationary phase, while a mixture of toluene/ethylacetate/FA (2:7:2, *v*/*v*/*v*) was used as the mobile phase. The plate was saturated for 30 min at 25 ± 2 °C and subsequently allowed to dry at RT. The bands separated on the HPTLC plates were scanned at a wavelength of 200–400 nm, with a maximum absorbance at a wavelength of 280 nm, finding 25.05 mg/g of GA in the PEE extract. Another study by Parimala and Shora [97] employed HPTLC as an analytical technique for the quantification of GA and other compounds in the hydroalcoholic extract of *Nymphaea nouchali* seeds. Samples were applied on GF_254_ silica gel plates, and a mobile phase composed of chloroform/ethylacetate/FA (2.5:2:0.8, *v*/*v*/*v*) was used. A densitometric scan was performed at 280 nm, and 0.27% GA was found in the extract. In addition, the HPTLC analysis revealed the presence of catechin (3.06%) and quercetin (0.04%) in the *N. nouchali* seed extract. These compounds are known for their antimicrobial and antioxidant properties, supporting the traditional use of the plant for the treatment of infections [92].

### 3.2. Capillary Electrophoresis (CE)

CE has been offered as an alternative to the traditional chromatographic separation methods (e.g., LC, GC, or (HP)TLC) used for the determination of phenolic compounds in plant extracts for the separation of phenolic acids [93]. In particular, CE is especially suitable for the separation and quantification of polar and charged compounds of low to medium molecular weight [98]. In addition, other related techniques have been developed from CE, such as zone capillary electrophoresis (CZE), micellar electrokinetic chromatography (MEKC), and capillary electrokinetic chromatography (CEC), usually coupled with UV or Ms detection [92]. All these techniques are based on similar principles to CE but differ in certain specific aspects, which allow them to be adapted to different types of analysis and to improve separation and detection. In this regard, Hemwech et al. [99] examined the efficiency of two types of capillaries (silica-coated capillary and fused silica capillary) used in two different separation modes (CEC and MEKC) to analyze seven phenolic acids and caffeine in tea samples. The results of the study showed that the use of capillaries coated with a silica layer in both modes (CEC and MEKC) was more efficient for tea analysis, providing fast and reproducible results. Specifically, the results of the CEC-UV analysis using silica-coated capillaries found 2.6 ± 0.1 mg/g, 3.9 ± 0.1 mg/g, and 4.8 ± 0.4 mg/g GA in three tea samples. There are a few more examples of CE used to separate and measure the amount of GA in plant material. However, the technique has some limitations compared to HPLC or GC, such as a low sensitivity in terms of solute concentration and poorer reproducibility. This is because the capillary that is employed as a detection cell has a short optical path length, and it can only accommodate small volumes [98].

### 3.3. Spectroscopic Techniques

#### Nuclear Magnetic Resonance (NMR)

**Figure 3:**
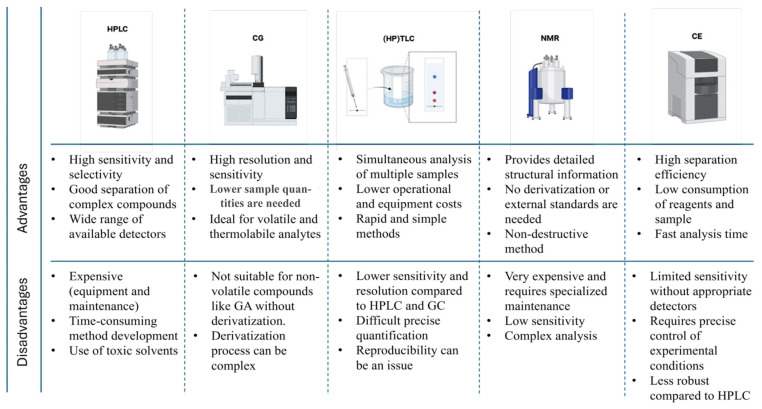
Advantages and drawbacks of the techniques used in GA analysis.

NMR spectroscopy is a method mainly used in the elucidation of molecular structures. ^1^H, ^13^C, or high-resolution magic-angle spinning (HR/MAS) NMR spectra can provide a wealth of chemical information on liquid and even semi-solid samples. In fact, NMR spectra of plant samples can act as fingerprints used to compare, discriminate between, or classify samples [98]. Yuan et al. [83] used ^1^H-NMR spectroscopy for the chemical characterization of commercial green teas. The authors identified and quantified the specific signals of GA and other phenolic compounds in the ^1^H-NMR spectrum, finding 0.34 to 1.88 mg/g GA in the different samples. In addition to GA (δ 7.14), they also identified (−)-epigallocatechin (δ 6.59), EGCG (δ 6.62), and caffeine (δ 7.68). On the other hand, Amargianitaki and Spyros [100] made an exhaustive summary of the research work related to the applications of NMR spectroscopy, focusing specifically on the use of two-dimensional nuclear magnetic resonance spectroscopy (2D NMR) to obtain the metabolic and/or non-volatile phenolic profile of port wines (Portugal). The studies reported the presence of several phenolic compounds, among them GA (δ 7.12). Although NMR has several advantages, such as the high structural elucidation power of the technique, it also has some drawbacks. The first (and most prohibitive) limitation is the high cost of the equipment. The second limiting reason is the relatively low sensitivity of NMR compared to other techniques such as HPLC or GS. Figure 3 shows the main advantages and disadvantages of each of the mentioned techniques, including NMR.

A solution to this weakness lies in the coupling of chromatographic techniques such as HPLC with NMR (HPLC-NMR) or NMR with detectors such as UV or MS (NMR-UV/MS), which enhances their analytical capabilities and provides a more complete and detailed view of the analyzed samples [101].

### 3.4. Other Techniques

Other chromatographic techniques have been used to purify and separate GA from complex matrices of phenolics and other compounds. In this regard, there are studies that have examined the presence of GA using ultraviolet–visible absorption spectroscopy (UV–Vis) [85], electrospray ionization mass spectrometry and tandem analysis ESI-(MS/MS) [86], and diffuse reflectance spectroscopy (DRS) [87], among others. These techniques provide an additional complementary methodology for the analysis of GA based on their intrinsic properties, thereby allowing for the performance of research studies on a variety of sample types, including both liquid and solid samples.

## 4. Biological and Therapeutic Properties

The biological properties of GA are extensive. It can act as an antibacterial, antioxidant, anticancer, antiviral, anti-allergic and anti-inflammatory agent. It has also been found to be effective in cardiovascular and metabolic diseases such as obesity, diabetes, and degenerative diseases [102]. Some of these properties and their mechanisms of action will be described in the following sections.

### 4.1. Antibacterial Activity

**Figure 4:**
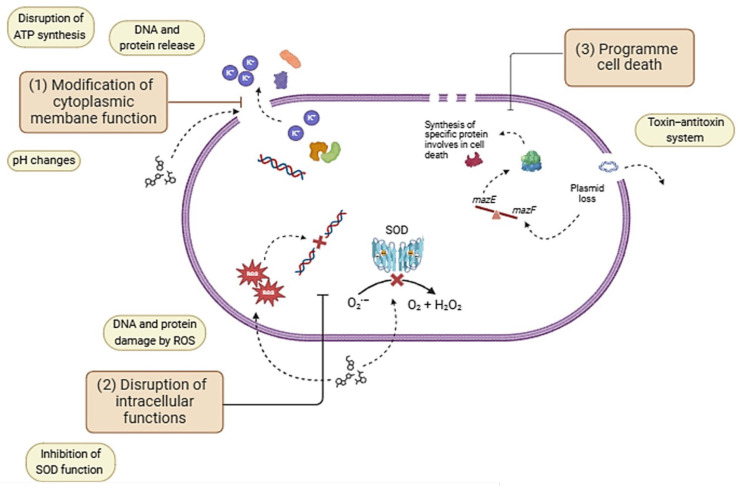
Main mechanisms of antimicrobial action of polyphenols on bacteria.

Numerous studies have reported the antibacterial potential of GA (see Table 2), although, in some of them, the exact mechanism of antimicrobial action is not known or not fully explained [103,104,105]. Nevertheless, it has been suggested that the mechanisms of action of polyphenols with antimicrobial properties are based on different factors at the cellular level, which may be individual or synergistic: (1) the modification of cytoplasmic membrane function; (2) disruption of intracellular functions; and (3) programmed cell death (PCD) [106,107,108,109]. The main antibacterial mechanisms of polyphenols are summarized in Figure 4.

#### 4.1.1. Modification of Cytoplasmic Membrane Function

One structural element that can sustain damage and lose its ability to function is the cytoplasmic cell membrane. Antimicrobial agents can cause damage to this membrane. As an example, a reliable marker of membrane damage is the release of components within cells. Small molecules, such as potassium ions, can impact the bacterial membrane when exposed to antimicrobial drugs, while large molecules (such as proteins, DNA, or RNA) may escape, leading to bacterial death [106]. In this regard, Lee and Je [110] studied how a treatment of chitosan grafted with GA affected the integrity of the cytoplasmic membrane of *E. coli* (Gram-negative) and *S. aureus* (Gram-positive). Gram-negative bacteria have both an outer membrane (OM) and inner membrane (IM) while Gram-positive bacteria have only an IM [111]. The main function of the OM in bacteria is to provide an additional protective and regulatory barrier against the external environment. When the OM is damaged by interaction with antimicrobial agents, 1-N-phenylanphthylamine (NPN, a fluorescent substance incorporated in the membranes) penetrates inside the bacterium, so that NPN can be used as an indicator of increased cell membrane permeability [112]. Based on this principle, the authors reported an increase in fluorescence by the addition of GA-grafted chitosan to the *E. coli* bacteria. A higher OM permeability is directly related to the IM in several ways, including exposure to toxic agents, disruption of the electrochemical gradient (adenosine 5′-triphosphate (ATP) synthesis), and the alteration of vital cellular functions [113]. Thus, as a consequence of OM permeation, the presence of GA-grafted chitosan also influenced the IM of *E. coli*. These results were similar to those observed for the IM of *S. aureus*, which allowed the authors to conclude that GA could bind to membrane components of both bacteria, leading to membrane permeabilization and the release of intracellular components [110]. Another study by Tian et al. [114] examined the mechanism of membrane damage by GA in *Yersinia enterocolitica*, in order to explore whether the use of GA could prolong the shelf life of pork. For this purpose, the intracellular ATP concentration (ATP_in_) and intracellular pH (pH_in_) were taken as markers of membrane integrity. Under normal conditions, the ATP_in_ level in cells remains stable. However, alterations in intracellular material balance and membrane integrity can lead to changes in ATP_in_. In this context, the authors observed that ATP_in_ was reduced after GA treatment, due to variations in cell morphology. Similarly, damage to the cell membrane has been reported to reduce the ability of bacteria to regulate their internal pH, leading to the denaturation of essential proteins and enzymes and the disruption of critical cellular functions. The results showed that GA solutions with MIC and 2 × MIC concentrations significantly reduced the pH_in_ of *Y. enterocolitica* from 8.50 (control) to 6.64 and 4.50, respectively. In addition, a field emission gun scanning electron microscopy (FEG-SEM) analysis was carried out, which confirmed that GA could destroy the membrane integrity of *Y. enterocolitica* bacteria.

#### 4.1.2. Disruption of Intracellular Functions

Many researchers have attributed the antibacterial activity of polyphenols to the alteration of the essential internal functions of the bacteria. When bacteria are exposed to polyphenols, reactive oxygen species (ROS) are generated, inducing endogenous oxidative stress in the bacteria. This oxidative stress can damage vital components of bacterial cells, which contributes to the antimicrobial activity of polyphenols [106]. In this context, Zhu et al. [115] investigated the antibacterial effects and mechanisms of GA on *E. coli* before and after treatment with UV-C light (UVC-GA). The study was based on the fact that GA has been found to be a compound that can behave as a photosensitizer. This means that during treatment with UV-C light, the antibacterial properties of GA are enhanced, because it forms quinone intermediates and ROS, which interact with the proteins and DNA of the bacteria, damaging it and contributing to its death [106]. In the study, the antibacterial effects were assessed through various metrics, including MIC, MBC, killing time curve, and cell membrane damage, as well as DNA and protein damage and biofilm formation. The results revealed that both GA and UVC-GA showed antibacterial properties against *E. coli*. For a deeper compression, GA and UVC-GA antibacterial mechanisms were compared by the real-time quantitative polymerase chain reaction (RT-qPCR) analysis of gene expression, primarily focusing on genes related to the two-component system (TCS) (*qseBC*, *basSR*, *phoPQ*, and *kdpDE*) and quorum sensing (QS) system (gene *luxS*). TCSs are signaling systems present in bacteria that allow them to respond to changes in their environment in different ways: interacting with the QS system to regulate virulence (*qseBC*), regulating outer membrane permeability (*basSR*), regulating resistance to environmental and antimicrobial stress (*phoPQ*), and influencing the response to osmotic stress (*kdpDE*) [116,117,118]. Moreover, QS is a bacterial communication mechanism that allows the coordination of group activities as a function of cell density. For example, the *luxS* gene is involved in the synthesis of the autoinducer AI-2, which influences the regulation of virulence, biofilm formation, and antibiotic resistance [119]. The analysis of both molecular mechanisms led to the conclusion that UVC-GA and GA have totally different inhibitory mechanisms on *E. coli*, mainly because the combined use of UVC and GA light has a stronger antimicrobial effect against this bacterium. The use of UVC light not only enhances the action of GA but also introduces its own bactericidal effects, resulting in greater efficacy in the inhibition of bacterial growth. Similar conclusions were obtained by Wang and coworkers [120] who observed an enhancement of the antibacterial activity of GA and propyl gallate against *E. coli* under UV-A light. The explanation for this phenomenon is based on the increased uptake of GA by bacteria when they are exposed to UV-A light. Once GA is internalized, the interaction between GA and UV-A induces the intracellular formation of ROS, leading to oxidative damage. Moreover, this interaction between GA and UV-A inhibits the activity of superoxide dismutase (SOD), which is responsible for catalyzing the conversion of the superoxide radical into less reactive species (H_2_O_2_ and O_2_). The combination of these stresses impacts bacterial DNA and metabolism, which ultimately leads to bacterial death.

**Table 2:** Summary of studies on the antimicrobial potential of GA.

Sample/Matrix Type	Bacterial Strains	Antibacterial Assay	Results	Mechanism of Action	References
GA-grafted chitosan	*Staphylococcus aureus* *Bacillus subtilis* *Bacillus cereus* *Enterococcus faecalis* *Listeria monocytogenes* *Escherichia coli* *Klebsiella pneumoniae* *Pseudomonas aeruginosa* *Salmonella typhimurium* *Shigella flexneri*	Broth dilution method Time–kill experiment against *E. coli* and *S. aureus* OM and IM permeabilization assay	MIC values from unmodified chitosan are 64–128 μg/mL against Gram-positive bacteria and 512–1024 μg/mL against Gram-negative bacteria while MIC values from GA-grafted chitosan are 16 to 64 μg/mL against Gram-positive bacteria and 128 to 512 μg/mL against Gram-negative bacteria. GA-grafted chitosan (I) at MIC suppressed both *E. coli* bacterial growth for 24 h and *S. aureus* bacteria. Moreover, over the MIC values, no viable cells were observed. OM and IM permeabilization experiments indicated that GA-crafted chitosan influenced the integrity of the membrane	Disruption of the cell membranes by GA-crafted chitosan. *S. aureus* and *E. coli* cells undergo cell membrane damage resulting in the release of their cellular components into the surrounding environment, becoming finally empty	[110]
3D chitosan–GA complexes	*E. coli* *S. aureus*	Broth dilution method	3D chitosan–GA complexes demonstrated a higher antimicrobial capacity than 3D chitosan alone, with an inhibition percentage of around 83% more than 3D chitosan alone, regardless of the bacterial strain and concentration used, indicating that the adsorption of GA effectively increases the antimicrobial activity of 3D chitosan	ND	[103]
Native pectin (Na-Pe) acylated with GA (Ac-Pe)	*E. coli* *S. aureus*	OD method	The inhibition rate of the pectin against *E. coli* and *S. aureus* improved from 2.93% and 8.92% (Na-Pe) to 26.95% and 42.18% (Ac1-Pe) and 31.56% and 47.87% (Ac2-Pe), respectively	ND	[104]
GA-loaded ovalbumin (OVA)–chitosan (CS) nanoparticles	*Morganella morganii* *E. coli*	Plate count method	The number of *M. morganii* was 1.7 × 10^9^ CFU/mL for pectin film, 8.7 × 10^8^ CFU/mL for GA–pectin film, 5.5 × 10^8^ CFU/mL for pectin film with OVA/CS nanoparticles, and 3.2 × 10^8^ for GA-loaded OVA/CS nanoparticles. On the other hand, compared with pure pectin film (2.7 × 10^10^ CFU/mL), the pectin film with GA-loaded OVA/CS nanoparticles (5.8 × 10^9^ CFU/mL) could retard the growth of *E. coli* Moreover, the quantity of histamine (toxic compound produced during food spoilage) was also measured, showing that the growth rate of this amine in salmon fillets treated with the pectin coating with GA-loaded OVA/CS nanoparticles was the lowest (51.6%) compared with the control group (140.0%)	ND	[105]
Chitosan (Ch) and zinc oxide nanoparticle-loaded gallic acid films, (Ch-ZnO@gal	*B. subtilis* *E. coli*	Agar well diffusion assay	The results of the antibacterial activity of Ch-ZnO@gal revealed that the antimicrobial activity is linearly related to the amount of GA in the nanoparticles, Ch-ZnO@gal3 (70 mg of ZnO@gal) being the most efficient film against both *B. subtilis* and *E. coli* bacteria	The released ROS from the ZnO@gal, together with Zn^2+^ ions, attack the negatively charged cell wall, which leads to leakage and ultimately death of bacteria	[121]
PVDF-grafted GA (PVDF-g-PGAL)	*S. aureus* *E. coli*	Plate growth inhibition assay	M0 (unmodified PVDF) showed minimal or no inhibition zones, indicating no antibacterial activity. In contrast, M3 (PVDF-grafted GA) showed clear zones of inhibition around the membrane, indicating antibacterial activity against *E. coli* and *S. aureus*. Furthermore, M3-SO_3_Na (PVDF-grafted GA + sodium sulphonate) showed higher inhibition zones compared to M3, due to the increased hydrophilicity of the membrane	The hydrophilic membrane reduces the chances of bacterial colonies establishing themselves and proliferating on its surface. In addition, hydrophilic surfaces attract water molecules, creating a thin aqueous layer on the membrane surface. This layer can inhibit bacterial adhesion and biofilm formation, as bacteria prefer to adhere to dry surfaces	[122]
Commercially obtained GA	*K. pneumoniae*	Broth dilution method	*K. pneumoniae* growth was reduced at 5 and 10 mM GA concentrations, but not at 2.5 mM	GA may affect the iron availability in *K. pneumoniae*, thus possibly repressing the *cps* transcription (the inhibition in the production of capsules reduces bacterial virulence)	[123]
Commercially obtained GA	*E. coli* *P. aeruginosa* *S. aureus* *L. monocytogenes*	Broth dilution method, physicochemical characterization of the bacterial surfaces	GA had antimicrobial activity against the bacteria tested, with a MIC of 1500 mg/mL for *E. coli*, 500 mg/mL for *P. aeruginosa*, 1750 mg/mL for *S. aureus*, and 2000 mg/mL for *L. monocytogenes.* In addition, GA also had bactericidal activity due to the MBC values for GA: 5000 mg/mL for *E. coli*, 500 mg/mL for *P. aeruginosa*, 5250 mg/mL for *S. aureus,* and 5500 mg/mL for *L. monocytogenes*	GA led to irreversible changes in membrane properties (charge, intra- and extracellular permeability, and physicochemical properties) through hydrophobicity changes, a decrease in negative surface charge, and the occurrence of local ruptures or pore formation in the cell membranes, with a consequent leakage of essential intracellular constituents	[124]
Commercially obtained GA	*S. flexneri*	- Broth dilution method - Time-dependent killing assay Antibacterial assays to elucidate the mechanism of action: viability assay, integrity of cell membrane, FESEM analysis	- GA showed a MIC value of 2 mg/mL and MBC value of 8 mg/mL against *Sh. Flexneri.* It showed that GA led to inhibitory effects, which was evidenced by reduced cell viability, destroyed cell membranes, and changes in bacterial morphology	GA effectively inhibited *Sh. flexneri* activity and its biofilm formation by regulating the expression of the mdoH gene and the OpgH protein (mutations in mdoH that affect OpgH function may reduce bacterial virulence)	[125]
Commercially obtained GA	*Proteus* spp. *E. coli* *Pseudomonas* spp. *Salmonella* spp. *Streptococcus* spp. *S. aureus*	Bacterial growth inhibition assay with OD measurement Petri dish biofilm assay Measurement of cell biomass concentration and EPS quantification	Different concentrations (1–200 mg/L) of GA showed antimicrobial effects by reducing the growth of single and multispecies bacteria (12–86%). Higher concentrations (100–200 mg/L) of GA had prominent inhibitory effects on biofilm formation. Further, GA (20–200 mg/L) exhibited a 93.43% biomass reduction and 88.6% EPS (polysaccharide) reduction	GA can reduce biofilm formation and EPS, where it is suspected to be the major reason of biofilm development	[126]
Commercially obtained GA	*Streptococcus mutans*	Broth dilution method Antibiofilm assays (pH drop test and proton permeability test)	GA showed a MIC value at 250 μg/mL, although GA did not inhibit the adherence of *S. mutans* in sub-MIC values. Regarding the antibiofilm assay, GA showed antimicrobial activity, reducing the number of viable cells (${1.46\times 10}^{9}$ vs. ${1.53\times 10}^{12}$). Moreover, GA sensitized the cells to acidification, thereby reducing the glycolysis and acid production in the biofilm	The biofilms treated with GA showed a different architecture–structure: less compact and less dense due to a reduction in the synthesis of glucans (produced by a downregulated expression of gtfB, gtfC, and gtfD genes in the biofilms). These changes in the biofilm structure occurred because of the bactericidal activity, reduction of soluble alkaline glucans, and acidogenicity of *S. mutans* by GA	[127]
Commercially obtained GA	*Paenibacillus larvae*	Microdilution method Agar well diffusion assay Spore germination assay	The MIC and MBC values of GA against *P. larvae* were 125 and 250 μg/mL GA (200 mg/mL) produced an average inhibition halo of 17.7 ± 0.39 mm against *P. larvae* in the agar well diffusion assay. In the presence of 125 and 250 μg/mL GA, spore germination rates were reduced to 83.9% and 18.7%, respectively	GA resulted in the leakage of proteins and nucleic acids (vital intracellular components of the bacteria), leading to bacterial death. Moreover, GA-mediated membrane and intracellular damage, together with its capacity for restricting biofilm formation, increase bacterial susceptibility to other antibacterial agents and eventually cause lethal effects	[128]

Abbreviations: No data (ND); minimum inhibitory concentration (MIC); minimum bactericidal concentration (MBC); outer membrane (OM); inner membrane (IM); Transmission Electron Microscopy (TEM); optical density (OD); reactive oxygen species (ROS); Field Emission Scanning Electron Microscope (FESEM).

#### 4.1.3. Programmed Cell Death (PCD)

In multicellular organisms, programmed cell death (PCD) is a crucial mechanism that culminates in cell suicide. Apoptosis, the traditional type of PCD in eukaryotes, is typified by DNA fragmentation and membrane depolarization. In contrast, in bacteria, the concept of PCD is not fully understood as in multicellular organisms, but it is known that there are mechanisms of cell death that can be triggered by different stimuli. In this context, the best-studied PCD systems are mediated by the toxin–antitoxin (TA) and apoptosis-like death (ALD) systems [108]. Two genes, one encoding a stable toxin and the other an unstable antitoxin that obstructs the toxin’s fatal activity, make up TA systems. A bacterium experiences PCD when it loses a plasmid or other extrachromosomal material, because the unstable antitoxin breaks down more quickly than the more stable toxin [108]. For example, the *E. coli* chromosome contains the TA module *mazEF*, in which the *mazF* gene specifies the stable toxin MazF and the *mazE* gene specifies the labile antitoxin MazE. Various stress conditions, including DNA damage, induce *E. coli* MazF to generate an alternative translation machinery responsible for the synthesis of specific proteins involved in cell death [129]. The synthesis of this class of proteins results in cell death, but without the morphological changes typical of apoptosis. This second death pathway is mediated by the *recA*-*lexA* system and, like mazEF, is also triggered by severe DNA damage [130]. Although numerous studies have evaluated the antimicrobial potential of different compounds to induce PCD [131,132,133,134], to our knowledge, no studies have explored the antimicrobial potential of GA through these pathways. Nevertheless, there are studies that have analyzed and proved the potential of some GA derivatives, including octyl gallate [135] and EGCG and theaflavin3,3′-digallate [136], to induce PCD in *Vibrio parahaemolyticus* and *Clostridium perfringens* bacteria, respectively.

### 4.2. Antioxidant Activity

In humans, oxidative stress arises when there is an imbalance between the body’s antioxidants and free radicals. This imbalance can lead to cellular and tissue damage, contributing to the development of various diseases, including neurodegenerative diseases, cardiovascular diseases, diabetes, and cancer [137,138]. Likewise, ROS accumulation contribute to the deterioration of food by causing oxidative damage to lipids, proteins, and other biomolecules in food products [139,140]. GA, widely recognized for its potent antioxidant properties, plays a dual role in both the food industry and healthcare.

In the food industry, GA is extensively used as a potent antioxidant that prevents spoilage during various phases such as the preparation, preservation, and transportation of edibles. As a result, GA helps extend the shelf life and preserve the quality of food items [141]. Its mechanism of action involves two primary pathways: The Hydrogen Atom Transfer (HAT) mechanism and the Single Electron Transfer (SET) process. In the HAT mechanism, GA donates a hydrogen atom, thereby neutralizing free radicals that contribute to oxidative damage. Alternatively, in the SET process, GA functions as an electron donor, effectively reducing oxidative stress by stabilizing reactive species. During these processes, GA itself may become a radical, but it has the inherent capability to stabilize into a non-reactive, harmless state, ensuring its effectiveness as an antioxidant [42,139]. GA plays also an important role in preserving the stability of lipid-containing foods by inhibiting lipid peroxidation. This function is accomplished through chelating metal ions, such as iron and copper. By binding to these metal ions, GA interrupts the chain reaction triggered by the free radical assault on unsaturated fatty acids [142].

Additionally, GA has been found to regulate the formation of 5-hydroxymethylfurfural (5-HMF) during food processing. This heat-induced contaminant is present in various foods and acts as an intermediate in the Maillard reaction (MR) and caramelization processes and is responsible for the flavor and color changes that occur. The mechanism underlying GA’s ability to prevent 5-HMF formation lies in its capacity to delay the oxidation of MR intermediates. Specifically, GA has been found to postpone the generation of free radicals from 1,4-pyrazine cation within model systems containing glucose and amino butanoic acids. This interference with the oxidation process disrupts the pathway leading to 5-HMF formation, thus reducing its presence in food products [143]. Moreover, GA has been reported to act synergistically with other antioxidants in food edibles, amplifying their combined antioxidant efficacy [141].

As previously mentioned, besides its food preservation capabilities, GA has shown considerable promise in protecting the human body from oxidative stress-related damage. In particular, GA can activate nuclear erythroid-2-related factor 2 (Nrf2), a crucial transcription factor that regulates the expression of antioxidant enzymes [102,144,145]. GA can also modulate various signaling pathways implicated in oxidative stress and inflammation, including nuclear factor-kappa B (NF-κB) pathways, by activating the Keap1/Nrf2/ARE pathway, leading to the expression of proteins that inhibit the IKK complex. This inhibition prevents NF-κB activation and lowers the inflammatory response [141,145,146]. At the same time, numerous protein kinases, like phosphoinositide 3 kinase (PI3K), protein kinase B (Akt), mitogen-activated protein kinase (MAPK), and 5 adenosine monophosphate-activated protein kinase (AMPK), are highly affected by GA. It is also important to note that by suppressing the MAPK pathway, GA reduces NF-κB activation, which in turn lowers the expression of pro-inflammatory cytokines and other mediators, thus decreasing inflammation and oxidative stress. Furthermore, GA’s activation of Akt and AMPK stimulates Nrf2, enhancing the expression of antioxidant enzymes [141,146]. Furthermore, the MAPK signaling cascades, inclusive of extracellular signal-regulated kinase (ERK), c-Jun N-terminal kinase (JNK), and p38 MAPK, play a pivotal role in diseases induced by oxidative stress. GA has been found to activate JNK and ERK, consequently improving oxidative damage. By activating JNK, GA can enhance cellular stress response mechanisms, helping cells to better manage and repair oxidative damage [141,147].

In addition, GA’s protective effects extend beyond this, to preventing glyoxal-induced injury. Glyoxal is a type of advanced glycation end-product (AGE) precursor, and its accumulation in biological systems can lead to various harmful effects. GA can directly inhibit the formation of AGEs by reacting with glyoxal and other dicarbonyl compounds. This prevents the glycation of proteins, which is a key mechanism of glyoxal-induced injury. Some studies additionally suggest that GA may help break existing AGE cross-links in proteins, thereby restoring their normal function and reducing cellular dysfunction [148].

Moreover, GA emerges as a promising protective agent against various forms of kidney damage induced by environmental toxins, drugs, and chemical exposure. GA has been found to interfere in this process by boosting the activity of the body’s natural defense mechanisms, particularly its antioxidant enzymes. Notably, bisphenol A (BPA), which is an endocrine disruptor molecule extensively used in plastics, is known to damage various body systems, particularly the kidneys. Research has revealed that GA can significantly restore the antioxidant system in the kidneys of BPA-treated rats, demonstrating its potential to counteract BPA-induced kidney toxicity [149]. Similarly, paraquat (PRQ), a toxic herbicide, poses severe health risks through ingestion, inhalation, or dermal exposure and is linked to multiple toxic cascades, ultimately leading to neuronal degeneration. GA has proven effective in preventing PRQ-induced kidney damage in mouse studies by elevating levels of vitamin C, SOD, and CAT, while remarkably decreasing serum protein carbonyl (PC) and MDA levels [150]. In the same context, cyclophosphamide (CP), commonly employed in the treatment of cancer and autoimmune diseases, has been identified as nephrotoxic in humans because it produces reactive oxygen species (ROS). GA prohibited CP-induced kidney toxicity in mice by elevating GSH and enhancing the activities of SOD, GPx, and CAT [141]. In another study involving rats with hypoxic-ischemic brain damage, GA was shown to reduce neuroinflammation and neuronal loss by inhibiting the production of ROS and proinflammatory cytokines [151]. Additionally, GA has been shown to inhibit the activation of oxidative stress by various chemicals and drugs, such as diclofenac, paclitaxel, ketamine, valproic acid, and ethanol, which are known to cause significant neurotoxicity after a long and unlimited exposure [141,152].

GA has also emerged as a promising candidate to prevent neurological disorders linked to oxidative stress, owing to its multifaceted mechanisms of action. Numerous studies have highlighted GA’s capacity to combat oxidative brain damage by boosting the body’s own antioxidant defense system and decreasing lipid peroxidation [153]. In fact, GA demonstrates neuroprotective effects against excitotoxicity induced by glutamate, attributed to its ability to modulate antioxidant profiles and inhibit proinflammatory cytokine generation [141]. Moreover, in the context of liver fibrosis progression, which can result in irreversible cirrhosis, GA demonstrated significant antioxidant and antifibrotic properties. This efficacy is attributed to its ability to reduce serum liver enzymes, enhance the expression of antioxidant enzymes, and suppress pro-inflammatory cytokines, positioning it as a promising natural treatment for liver fibrosis [154]. Additionally, GA has been shown to reduce inflammatory mediators and increase antioxidant enzymes in non-alcoholic fatty liver disease (NAFLD), a common liver condition that can lead to chronic liver disease and significant oxidative stress [146]. Likewise, in studies using animal models, GA has been found to alleviate liver fibrosis caused by thioacetamide exposure, by activating hepatic antioxidant enzymes and reducing levels of malondialdehyde (MDA), a marker of oxidative stress [141]. GA can also protect against inflammation-induced liver damage by improving the expression of key proteins involved in antioxidant defense, such as NF-κB, Nrf_2_, and HO-1, while enhancing overall liver antioxidant capacity [141,155].

In diabetes, a long-term metabolic condition often involving shifts in hormones and neurochemicals, GA has been found to decrease lipid peroxidation and elevate GSH levels in the hippocampus and prefrontal cortex of diabetic rats induced by streptozotocin [156]. In another study conducted on mice, the antioxidant activity of GA was explored in relation to diabetes. The research found that diabetes negatively impacts the male reproductive system, leading to degenerative changes in the testes and epididymis. However, GA significantly boosted natural antioxidant defenses and reduced methylglyoxal-induced inflammation in these organs [157].

Furthermore, GA has been shown to reverse the hepatorenal dysfunction caused by aflatoxin B1 (AFB1) by increasing GSH levels and reducing ROS. Additionally, when combined with monoisoamyl-dimercaptosuccinic acid, GA can counteract arsenic-induced damage in red blood cells by mitigating oxidative and nitrosative stress [158]. Further research has also revealed that GA significantly lowers harmful compounds like MDA and NO, while increasing beneficial antioxidants like GSH and the activities of GPx and SOD in the heart tissues of rats exposed to sodium arsenite [159]. GA can also improve heart function in rats with ischemia/reperfusion injury by enhancing antioxidant defenses and reducing the cell damage caused by isoproterenol in hypertrophic hearts [160]. In another study involving mice with induced myocardial hypertrophy and dysfunction, GA effectively reduced harmful superoxide levels. Additionally, GA showed protective effects against heart damage caused by the chemotherapy drug doxorubicin by lowering MDA levels and boosting antioxidant enzyme activities [141]. In the same context, Table 3 summarizes the antioxidant benefits of GA based on studies conducted exclusively on mice. These studies highlight GA’s efficacy in enhancing antioxidant enzyme activities, reducing oxidative stress markers, and providing protective effects against different forms of oxidative damage.

### 4.3. Anticancer Mechanisms of GA

In terms of its anti-cancer effects, GA has been observed to disrupt multiple signaling pathways crucial for cancer cell survival, encompassing cell cycle regulation and the modulation of oncogene expression. Additionally, GA induces oxidative stress, activates the mitochondrial apoptotic pathway, inhibits angiogenesis, and modulates key signaling pathways such as PI3K/Akt/mTOR (mammalian target of rapamycin) and MAPK/ERK suppressing tumor growth [141,180,181]. The last activity of GA is critical, as the PI3K/Akt/mTOR and MAPK/ERK signaling pathways are vital in regulating cell growth, survival, and proliferation. The PI3K enzyme initiates a cascade of events that promote cell growth and metabolism, with Akt serving as a key mediator of cellular processes such as proliferation and survival. Meanwhile, mTOR, which forms two complexes known as mTORC1 and mTORC2, plays a crucial role in regulating protein synthesis, cell growth, and metabolism. The overactivation of mTOR, in particular, is implicated in cancer cell proliferation and resistance to apoptosis [181]. Similarly, the MAPK/ERK pathway transmits signals from the cell surface to the nucleus, governing cellular processes like proliferation and differentiation. The dysregulation of MAPK/ERK signaling can lead to uncontrolled cell growth and contribute to tumor formation [180].

Furthermore, GA has been found to target two key efflux transporters, P-glycoprotein (P-gp) and breast cancer resistance protein (BCRP), inhibiting them and increasing systemic drug exposure. Linagliptin, a DPP-4 inhibitor for type 2 diabetes, is a P-gp substrate, and its absorption is greatly affected by P-gp’s efflux action. A seven-day pretreatment with GA resulted in a 2.2-fold increase in linagliptin’s permeability in the ileum [182]. Similarly, rosuvastatin, a known BCRP substrate, showed a 2.34-fold increase in oral exposure when co-administered with GA (10 mg/kg) compared to the control group, along with a significant 39.2% reduction in cumulative biliary excretion over 8 h, indicating that GA inhibits rosuvastatin transport into bile, thereby increasing its plasma exposure [183]. Additionally, GA has been found to significantly inhibit the organic anion-transporting polypeptide (OATP1B3) and reduce the transport functions of organic anion transporter 2 (OAT2), organic anion-transporting polypeptide 2B1 (OATP2B1), and organic cation/carnitine transporter 2 (OCTN2) in human embryonic kidney 293 (HEK293) cells overexpressing these solute carrier (SLC) transporters. These findings suggest that the co-administration of GA with therapeutic drugs could result in potential drug interactions due to competitive cellular uptake in hepatocytes [184].

Numerous studies have also indicated that GA and its derivatives exhibit anticancer effects across a variety of cancer types. These include prostate cancer, melanoma, leukemia, oral cancer, colon cancer, lymphoma, and breast cancer cells [102,185,186]. In glioblastoma cell-bearing rats, GA resulted in a 90% reduction in tumor volume and reduced oxidative damage in the brain [187]. In prostate cancer cells, GA decreases the survival of DU145 cells by inducing ROS, and by triggering mitochondria-mediated apoptosis. Additionally, GA causes cell cycle arrest in the G2/M phases by activating Checkpoint Kinase 1 (Chk1) and Checkpoint Kinase 2 (Chk2), both critical proteins for cell cycle control, while inactivating Cell Division Cycle 25C (Cdc25C), a phosphatase that activates cyclin-dependent kinases, and Cyclin-dependent Kinase 1 (Cdc2), an essential protein for the transition from the G2 phase to the M phase in the cell cycle [188]. Another investigation examined the antitumor activity of GA on PC3 prostate cancer cells. The study revealed that GA induces DNA damage and activates multiple DNA repair genes [189]. Furthermore, in PC3 cells treated with GA, there was a notable inhibition of the JNK, PKC, p38, and PI3K/AKT signaling pathways, which ultimately led to the blockage of MMP-2 and MMP-9 activity in these cells [185].

In A375S2 human melanoma cells, GA demonstrated a significant inhibition of cell proliferation and an induction of apoptosis. Following GA treatment, cell proliferation decreased in a dose- and time-dependent manner. The process of apoptosis involves a reduction of the anti-apoptotic protein B-cell lymphoma 2 (Bcl-2) and an increase in the pro-apoptotic B-cell lymphoma 2-associated X protein (Bax). GA lowers the mitochondrial membrane potential over time, causing the release of cytochrome c. This release activates caspase 9 and caspase 3, which leads to apoptosis. Furthermore, GA promotes the expression of endonuclease G (Endo G) and apoptosis-inducing factors (AIFs) [190]. Another study demonstrated the impact of GA on the gene expression and protein levels of matrix metalloproteinases (MMPs), as well as the in vitro migration of melanoma cells. The results showed that GA treatment led to a reduction in MMP signaling pathways and mRNA levels in A375S2 cells, indicating that GA functions as an antimetastatic agent. Additionally, GA influenced the Ras and phosphorylated extracellular signal-regulated kinase (p-ERK) signaling pathways, resulting in the suppression of MMP-2 in A375S2 melanoma cells [185]. Another survey explored the anticancer effects of GA on colon cancer cells. Treatment with GA resulted in DNA fragmentation and changes in cell morphology, suggesting that GA triggered apoptosis in these cells [191]. In a different study, the antitumor properties of GA on HCT-15 colon cancer cells were presented. Treatment with GA led to a dose-dependent reduction in cell survival. Notable changes included cell contraction, rounding, and detachment from the substrate compared to the control group [102].

Additionally, GA’s anticancer potential against HL-60 and HL-60RG promyelocytic leukemia, as well as K562 human leukemia cells, was studied. It was found that GA induces cell cycle arrest at the G0/G1 phase. This arrest is the result of the multiple mechanisms involved, including the inhibition of ribonucleotide reductase, DNA damage and fragmentation, cytochrome c release, the upregulation of Bcl-2 protein, AIFs, and Endo G, and the activation of caspase 4, 9, and 3. Furthermore, it was found that GA can inhibit the BCR/ABL tyrosine kinase, activate NF-κB, and enhance levels of Bax, Fas ligand, and p53 [192,193,194]. In addition, the treatment of HeLa cervical cancer cells with GA resulted in several effects. These included a depletion of GSH, reduction in mitochondrial membrane potential, downregulation of the EGFR, Erk/p-Erk, and Akt/p-Akt signaling pathways, activation of caspases, the cleavage of the poly (ADP) ribose polymerase, and cell cycle arrest in the G1 phase [195].

Moreover, GA has been found to enhance the efficacy of conventional chemotherapy and radiotherapy treatments [196]. In particular, the anticancer effects of GA, whether used alone or with cisplatin, a common chemotherapy drug, were observed in non-small-cell lung cancer (NSCLC). Additionally, pretreatment with low-level laser therapy (LLLT) followed by GA treatment on breast cancer and melanoma cells resulted in higher cell death rates compared to GA treatment alone [197]. However, chemotherapeutic drugs often entail severe side effects and foster drug resistance. To address these challenges, the integration of natural products with anticancer properties, such as GA, emerges as a promising strategy [198]. In the case of interleukin-8 (IL-8), a compound that acts as a survival factor for cancer cells and contributes to cancer metastasis, proliferation, and angiogenesis, inhibiting IL-8 is valuable in cancer therapy. GA, at concentrations of 300, 550, 670, and 800 μM, significantly reduces the proliferation of hepatocellular carcinoma (HCC) cells by downregulating IL-8. In fact, it is proposed that this reduction in HCC cell proliferation occurs through the induction of apoptosis, without notably affecting the cell cycle [199]. Additionally, GA has been found to interact with other molecular pathways involved in cancer progression. The downregulation of Epidermal Growth Factor Receptor (EGFR) and Proline, Glutamic Acid, and Leucine Rich Protein 1 (PELP1) can reduce hormone resistance in breast cancer cells to therapy. This is achieved through the interaction of PELP1 with Coactivator-Associated Arginine Methyltransferase 1 (CARM1) [200].

Furthermore, research has demonstrated that GA at concentrations up to 125 μg/mL can inhibit angiogenesis in glioma and cervical cancer cells by downregulating the signaling pathways of ADAM metallopeptidase domain 17 (ADAM17), Akt, ERK, and EGFR [196,201]. Another factor that plays a critical role in angiogenesis is VEGF. In cancer, VEGF and HIF-1α are closely related, with HIF-1α promoting VEGF to drive metastasis. GA can counteract cancer development by boosting PTEN expression, which negatively regulates Akt. This decrease in Akt expression leads to reduced HIF-1α levels, inhibiting VEGF, and consequently suppressing angiogenesis and metastasis [202].

Nevertheless, one of the significant challenges associated with GA is its poor bioavailability, primarily due to its rapid elimination and insufficient absorption. Nano-scale delivery systems offer substantial benefits in enhancing its bioavailability and therapeutic effectiveness by ensuring efficient encapsulation, protection from degradation, and targeted delivery. Various nano-carriers, such as gold nanoparticles, polymeric nanoparticles, dendrimers, and nanodots, have been used to deliver GA [141]. Notably, Au nanoparticles loaded with antitumor agents have been widely used in cancer therapy, due to their unique physicochemical properties, ease of synthesis, and high biocompatibility [203]. These characteristics make them ideal for applications in drug delivery, gene delivery, and imaging. More specifically, GA-loaded Au nanoparticles have proven very effective in breast cancer therapy by inhibiting cell migration through the downregulation of the p300 and NF-κB/c-Jun pathway. This leads to a reduction in EGF expression, subsequent downregulation of matrix metallopeptidase 9 (MMP-9), and suppression of breast cancer metastasis [204]. Moreover, GA-loaded Au nanoparticles suppress cancer cell proliferation by inducing apoptosis, underscoring the efficacy of Au nanoparticles as carriers for GA delivery [205]. Furthermore, Au nanoparticles can be designed for the co-delivery of GA with chemotherapeutic agents such as doxorubicin. Given that chemoresistance impairs the effectiveness of doxorubicin in cancer treatment, nanoparticles can enhance its delivery to tumor sites, thereby improving its antitumor activities [102,206].

GA-modified chitosan and caseinophosphopeptide nanoparticles were also created to deliver (−)-EGCG, showing strong antioxidant effects and heightened anticancer activity against Caco-2 colon cancer cells. Moreover, GA-conjugated chitosan effectively prevented lung metastasis in colorectal cancer models. Additionally, GA treatment in rats countered the adverse changes in enzyme levels caused by 1,2-dimethyl hydrazine-induced colon carcinogenesis, normalizing the balance between phase I and phase II enzymes [207]. In another study, anticancer nano-delivery systems using a graphene oxide–gallic acid (GOGA) nanocomposite were used. This GOGA nanocomposite showed strong anticancer activity against liver cancer (HepG2) cells, with the cytotoxic effects primarily attributed to the release of GA [208]. While numerous studies highlight the anticancer properties of GA in various human cells, rat models, and nanoparticle delivery systems, a select few are detailed in Table 4.

### 4.4. Antiviral Activity

GA has garnered significant attention for its antiviral properties. The research indicates that GA exhibits broad-spectrum antiviral activity, affecting various types of viruses through multiple mechanisms. One notable aspect of GA ‘s antiviral capability is its inhibition of viral replication. In a recent study, researchers have successfully converted GA into biocompatible graphene quantum dots (GAGQDs), which exhibit potent antiviral properties against the pseudorabies virus (PRV). The GAGQDs demonstrated a remarkable ability to suppress PRV proliferation, achieving a 10,000-fold reduction in viral titers. Investigation into the antiviral mechanism of GAGQDs revealed their efficacy in inhibiting the adsorption, invasion, and replication stages of PRV infection. Treatment with GAGQDs alters the expression levels of interferon-associated antiviral proteins, including mitochondrial antiviral-signaling protein, signal transducer and activator of transcription 1, and 2′,5′-oligoadenylate synthetase 1, implying that GAGQDs can activate innate antiviral immune responses, thereby amplifying antiviral efficacy. Additionally, treatment with GAGQDs mitigates clinical symptoms and lowers mortality rates in mice infected with PRV, showcasing the enhanced therapeutic efficacy of GAGQDs against PRV infection in both in vitro and in vivo settings. These findings suggest that GAGQDs could be a promising candidate for the development of novel antiviral therapies [37].

Additionally, sixteen GA derivatives were docked against five crucial non-structural proteins of SARS-CoV-2, which are considered promising targets for small-molecule inhibitors: nsp14, nsp13, nsp12, nsp5, and nsp3. The crystal structures of these proteins were obtained from the Protein Data Bank, while the 3D structures of the sixteen GA derivatives and three control drugs were sourced from PubChem [213]. Compounds demonstrating lower binding energies compared to the control drugs were identified and subsequently subjected to pharmacokinetic screening via the AdmetSAR server. The study’s results indicated that 4-O-(6-galloylglucoside) exhibited binding energy values of −8.4, −6.8, −8.9, −9.1, and −7.5 kcal/mol against Mpro, nsp3, nsp12, nsp13, and nsp15, respectively. The ADMET profile analysis indicated that 4-O-(6-galloylglucoside) undergoes liver metabolism and exhibits a very high affinity for plasma protein binding. These properties suggest its potential as an effective inhibitor against these SARS-CoV-2 proteins.

Moreover, GA’s antioxidant properties contribute to its antiviral activity. By reducing oxidative stress within host cells, GA helps maintain cellular integrity and function, creating an unfavorable environment for viral proliferation [214]. This multifaceted antiviral action makes GA a promising candidate for further development as a therapeutic agent in the treatment and prevention of various viral infections.

### 4.5. Anti-Alzheimer Activity

Alzheimer’s disease (AD) is a degenerative neurological condition marked by a progressive decline in symptoms as the disease advances. This condition leads to brain atrophy and the death of brain cells [215]. AD is the most common type of dementia and is linked to a significant cognitive decline that disrupts daily activities [216]. AD is characterized by the presence of abnormal neurofibrillary tangles and neuritic plaques. Neuritic plaques are spherical, microscopic lesions composed of an extracellular layer of Aβ-peptide, which forms due to the enlargement of axonal terminals. Beta-amyloid induces oxidative stress and triggers an inflammatory response, leading to neuronal damage and contributing to neurological disorders [217]. Recent research indicates a connection between metal dyshomeostasis and neurodegenerative amyloid diseases. It has been observed that Cu (II) ions bind to amyloid β fibrils, resulting in the production of reactive oxygen species [218]. An abnormal state of beta-amyloid 42 leads to the aggregation of amyloid, which in turn promotes neuronal damage and the loss of cholinergic neurons in the forebrain, commonly resulting in dementia [219]. The results of a study by Savelieff et al. (2014) indicate that specific compounds possess dual capabilities as metal chelators and interactors with amyloid aggregates. These findings suggest the potential for the development of novel multifunctional small molecules that exhibit enhanced activity and selectivity against toxicity and metal-induced amyloid formation [220]. Metal ions are crucial in the progression of neurodegenerative diseases, indicating that metal chelation therapy might be a promising therapeutic approach for treating these conditions. GA exhibits a dual inhibitory role: in addition to its known anti-amyloidogenic properties, it also effectively inhibits metal-induced aggregation. Spectroscopic analyses, including UV–Vis and fluorescence spectroscopy, confirm GA’s ability to chelate Mg^2+^ ions, thereby preventing metal-induced aggregation [221].

Current treatment approaches are inadequate in effectively preventing the symptoms of AD. The existing therapeutic paradigm for AD involves a combination of pharmaceutical and nonpharmacological strategies aimed at mitigating cognitive and functional decline [222]. Brain-derived neurotrophic factor (BDNF) regulates synaptic plasticity, neural differentiation, and cell death processes. BDNF has been detected in various regions of the brain, with particularly high concentrations in the hippocampus [223]. Furthermore, a correlation has been observed between low levels of BDNF and AD [224]. Elevated levels of BDNF enhance brain function. Trimethyltin (TMT) chloride, a toxin implicated in the advancement of AD, often leads to reductions in BDNF levels [225]. Tumor necrosis factor-alpha (TNF-α) significantly contributes to the central nervous system’s response to injury. Elevated levels of TNF-α are the primary risk factor for AD in individuals experiencing mild cognitive impairment [226]. In rats, an administration of GA at doses of 50 and 100 mg/kg elevated hippocampal levels of BDNF compared to rats exposed to TMT toxicity. GA also increased hippocampal levels of TNF-α, suggesting potential benefits for patients with AD [227]. GA administered at a dose of 30 mg/kg improved memory performance and passive avoidance, while also enhancing enzymatic and non-enzymatic functions such as CAT, GPx, and SOD activities in the hippocampal regions of rats with intracerebroventricular streptozotocin-induced AD’s disease. Additionally, GA administration reduced the levels of TBARS in these brain regions [228].

### 4.6. Anti-Inflammatory

Inflammation plays a crucial role in the pathogenesis of various chronic diseases, including autoimmune disorders, cardiovascular diseases, endocrine disorders, cancers, and neuropathic conditions [229]. GA has been observed to induce the acetylation of nuclear factor kappa B (NF-κB), leading to its stabilization and a subsequent reduction in cytokine production in microglial cells. This process helps shield neurons from the neurotoxic effects of amyloid beta (Aβ) [230].

GA exhibits anti-inflammatory properties, as demonstrated in a mouse model of acute inflammation induced by zymosan. In vitro studies have revealed that GA exerts its effects by interacting with leukocytes containing multiple nuclei. GA may inhibit the initiation of the inflammatory process by eliminating superoxide anions, suppressing the release and activity of myeloperoxidase, and potentially influencing the accumulation of active NADPH oxidase [231]. Some studies documented that GA effectively suppressed PGE-2 production and LPS-induced nitric oxide (NO), as well as interleukin-6 (IL-6) secretion, without exhibiting cytotoxic effects [232,233]. Sripanidkulchai and Junlatat [234] investigated the anti-inflammatory properties of *Phyllanthus emblica* Linn. extract, with GA identified as its principal component. GA demonstrated a dose-dependent inhibition of pro-inflammatory gene expression, including interleukin-1β (IL-1β), cyclooxygenase-2 (COX-2), IL-6, and inducible nitric oxide synthase (iNOS). The protective effect of GA against inflammatory damage has been demonstrated in various diseases and conditions in vivo, such as chronic obstructive pulmonary disease [235], obesity [236,237], cisplatin nephrotoxicity [238], colitis [239,240], diabetes [241], neuroinflammation [242], and infection [243]. Key mechanisms include reducing the expression of inflammatory mediators [235,237,239,243], inhibiting the phosphorylation or nuclear translocation of p65-NF-κB [235,239,240], suppressing the activation of signal transduction and transcription factors [240], and downregulating mRNA and protein expression [235,242].

The proposed pathophysiology of central nervous system neurodegenerative diseases, such as spinal cord injury, Parkinson’s disease, AD, and stroke, involves a vicious cycle of oxidative stress, the aggregation of protein, and cellular death [244]. Neuroinflammation is believed to be at the core of this cycle. The systemic administration of GA (100 mg/kg) markedly reduced the ED-1 (a marker of activated microglia), proinflammatory cytokine IL-1β, lipopolysaccharide (LPS)-induced elevation of glial fibrillary acidic protein (a marker of activated astrocytes), and the inducible iNOS in the substantia nigra of rat brains infused with LPS. GA mitigated α-synuclein aggregation, a hallmark of neurodegeneration in the central nervous system, and reduced the LPS-induced increase in heme oxygenase-1, a redox-regulated protein. These findings suggest that GA can inhibit the oxidative stress and protein aggregation induced by LPS. GA prevented the activation of caspase 3, a biomarker of programmed cell death, induced by LPS. Furthermore, GA prevented the LPS-induced elevation of receptor-interacting protein kinase (RIPK)-1 and RIPK-3 levels, which are biomarkers associated with necroptosis. These findings suggest that GA inhibits both necroptosis and apoptosis in the nigrostriatal dopaminergic system of LPS-exposed rat brains [242]. GA decreased NFκB, tenascin-C, the expression of COX-2, chondroitin sulfate proteoglycans, and glial fibrillary acidic protein in astrocytes within the LPC-induced inflammation model. Additionally, GA markedly increased the levels of myelin protein in both oligodendrocyte cell bodies and neurites [245]. Table 5 summarizes the anti-inflammatory effects of GA.

### 4.7. Anti-Diabetes

Diabetes mellitus is a persistent metabolic condition characterized by hyperglycemia caused by diminished insulin secretion and activity [246]. It is associated with several complications, including neuropathy, cardiovascular diseases (both microvascular and macrovascular), and nephropathy, primarily through its impact on the body’s antioxidant mechanisms [247]. Numerous studies have confirmed that excessive ROS production and oxidative stress are crucial factors in the progression of diabetes and its associated complications. This mechanism operates through modifications in lipid peroxide production, antioxidant enzyme levels, glucose auto-oxidation, glutathione metabolism, advanced glycation end product formation, and alterations in various signaling pathways in different tissues [248,249]. Numerous herbal and phytomedicine formulations have been evaluated in the quest to develop new antidiabetic drugs based on natural products. GA exhibits diverse bioactivities, including the inhibition of lipid peroxidation, the maintenance of the endogenous defense system, radical scavenging, the modulation of cell signaling pathways, and metal ion chelation [42]. Literature studies have shown that GA can mitigate the adverse effects of diabetes in rats by modulating biochemical and histopathological biomarkers of oxidative stress and influencing various cellular signaling pathways. GA also showed protective effects on pancreatic β-cells; it enhanced cellular glucose uptake, and increased both insulin sensitivity and plasma insulin secretion. Table 5 summarizes the antidiabetic effects of GA observed in both in vivo and in vitro test models.

**Table 5:** Other biological activities of GA.

Model	Condition	Main Findings	Reference
**Anti-inflammatory**			
Rats	In vivo	- Dose-dependent decreases in IL-6 and TNF-α levels.	[250]
Suppressing NF-κB signaling pathway in IPEC-J2 cells	In vitro	- In IPEC-J2 cells, GA pretreatment significantly decreased the elevated expression of tumor necrosis factor-α and interleukin-8 genes induced by LPS.	[26]
Control inflammation in NAFLD and NASH	In vitro	- By activating AMP-activated protein kinase (AMPK) in HepG2 cells, GA reduced the fat accumulation induced by palmitic acid.	[251]
Nrf2 antioxidant response element signaling pathway	In vitro and in vivo	- The PM10 groups exhibited a substantial increase in epithelial permeability and inflammatory markers. - Additionally, there was a notable reduction in the expression of Nrf2 and its upstream regulator genes.	[147]
Mice, suppressed interleukin-33 and group 2 innate lymphoid cells	-	- GA was found to lower IL-13 and IL-5 levels in bronchoalveolar lavage fluid (BALF) and to reduce IL-33 expression in lung tissue. This effect is accomplished by inhibiting MyD88 expression and downregulating NF-κB signaling pathways, leading to a reduction in IL-33 production.	[252]
Al_2_O_3_-induced myocardial injury	-	- ↓ CPK, LDH, CK-MB, MDA, LDL, TNF-α, and TG - ↑ SOD, HDL, CAT, and GSH	[253]
STZ-induced oxidative stress in testis of rats	-	↓ TNF-α, NOS2, VEGF, and MDA	[254]
**Antidiabetic**			
Mice, enhances insulin sensitivity and glucose transporters via Akt and PPAR-γ signaling pathways.	2–20 µM	- GA treatment enhanced insulin sensitivity by activating the Akt signaling pathway rather than the AMPK signaling pathway.	[255]
Mice, enhances lipid profile, glycemic and liver function	8.436 mg	- Promotes the repair of tissue damage in the pancreas and liver. - GA regulates autophagy in a diabetic cell model using Rin-5F cells.	[256]
Mice	50 mg/kg	- GA regulated lipid peroxidation (measured by TBARS) and antioxidant enzymes (GPX, superoxide dismutase, and catalase) in the liver and kidney, which are affected by diabetes-related complications caused by hyperglycemia.	[257]
- Mice 4- and 9-month-old groups - APPswe/PS1dE9 transgenic	30 mg/kg through gavage	- LTP - Aβ1–42 aggregation - Cognitive deficits - Expression of synaptic marker proteins	[258]
STZ-induced diabetic rat	20 mg/kg	- Reduces TNF-α levels, while increasing the upregulation of adiponectin and PPARγ mRNA.	[259]
Rat, Aβ hippocampal injection	50, 100, 200 mg/kg	- Hippocampal LTP	[260]
Pheochromocytoma12 cells	GA: Aβ 2.0: 1.0 M	- Toxicity - K-CN fibril formation	[261]
Mice, scopolamine-induced amnesia	10 mg/kg	- AChE activity - Transfer latency in the elevated plus maze (EPM) test - Duration spent in the target quadrant during the Morris water maze (MWM) test	[262]
Rat, i.p. injection of TMT 8 mg/kg	50, 100 mg/kg	- Hippocampal level of TNF-a - Hippocampal level of BDNF	[263]
Mice, via mTOR/PPARγ/AMPK signaling	3 mg	- Decreased expression levels of p-AMPK and increased levels of peroxisome proliferator-activated receptor gamma (PPARγ), LOX-1, NF-κB, COX-2, and p-mTOR.	[236]

### 4.8. Anti-Obesity

While GA has been known for its efficacy in treating conditions like hemoptysis since the 1800s [264], research highlighting its anti-obesity properties only began to surface approximately thirty years ago [265]. Using the keywords “GA and metabolic disease” in a search yielded about 246 articles. However, only 60 studies specifically explored the positive effects of GA on complications associated with obesity [266].

Using the experimental models mentioned earlier, GA has shown significant potential in alleviating various complications associated with obesity, as summarized in Table 2. Concise evidence indicates that GA effectively reduces body weight in obese rodents [267]. This reduction can occur either through the inhibition of lipid droplet formation in the liver and adipose tissue or by lowering serum levels of low-density lipoprotein and triglycerides. These effects have been observed in both cultured adipocytes and high-fat diet-fed rats [268,269]. The main mechanism behind GA’s therapeutic benefits is believed to be its ability to modulate lipid and glucose metabolism. The modulatory effects of GA on lipid and glucose intermediates likely contribute to its ability to improve glucose uptake [270], increase energy expenditure, and enhance insulin sensitivity [271]. GA’s ability to enhance insulin sensitivity may be attributed to its regulation of the PI3K/Akt signaling pathway. Additionally, its activation of the AMP-activated protein kinase (AMPK) could influence substrate metabolism, as demonstrated in multiple studies [272]. Moreover, natural compounds such as resveratrol and celastrol have been proven to regulate glucose and lipid metabolism, thereby alleviating obesity-related complications like inflammation and oxidative stress. These effects are primarily achieved through the modulation of mechanisms such as PI3K/Akt and AMPK [273].

Moreover, GA may mitigate obesity-related complications by increasing adiponectin levels and regulating genes involved in adipogenesis and cell proliferation [266]. For instance, GA can promote the browning of adipose tissue by upregulating the expression of peroxisome proliferator-activated receptor gamma (PPARγ) and activating the NAD-dependent deacetylase sirtuin-1 (SIRT1)/peroxisome proliferator-activated receptor gamma coactivator 1 alpha (PGC1α) pathway [272]. GA influences adipogenesis by increasing the protein expression of fatty acid synthase (FAS), activated caspase 3/9, tumor protein 53 (p53), and FAS ligand. Interestingly, GA can inhibit cholesterol synthesis by blocking β-Hydroxy β-methylglutaryl-CoA reductase activity, similar to the action of statin drugs [274]. However, some data compare GA to the antidiabetic agent pioglitazone [271].

### 4.9. Anti-Hypertensive

GA exhibits significant antihypertensive effects through multiple mechanisms. Research has demonstrated that GA can lower the blood pressure by improving endothelial function and enhancing nitric oxide production, which promotes vasodilation. Additionally, GA inhibits the activity of angiotensin-converting enzyme (ACE), a crucial regulator of blood pressure, thereby reducing the formation of angiotensin II, a potent vasoconstrictor. It also exerts antioxidant effects that mitigate oxidative stress, a contributor to hypertension. Furthermore, GA’s anti-inflammatory properties help reduce vascular inflammation, further contributing to its antihypertensive benefits. Collectively, these actions make GA a promising natural compound for managing hypertension.

Kang et al. [275] explored the vasorelaxant and antihypertensive properties of GA. The impact of GA on endothelium-dependent vasorelaxation was investigated in human umbilical vein endothelial cells. It was found that GA elevated nitric oxide (NO) levels by enhancing the phosphorylation of endothelial nitric oxide synthase (eNOS). This NO production effect was diminished when the cells were pretreated with the eNOS inhibitor NG-nitro-l-arginine methyl ester. Additionally, the antihypertensive effect of GA was investigated through its inhibition of ACE, with an observed half-maximal inhibitory concentration (IC_50_) of 37.38 ± 0.39 μg/mL. In silico simulations indicated that GA has a binding energy of −270.487 kcal/mol when it binds to the active site of ACE. Additionally, in spontaneously hypertensive rats (SHRs), GA significantly reduced their blood pressure, demonstrating effects similar to those of captopril. These findings indicate the potential of GA, derived from *Spirogyra* sp., as a treatment for cardiovascular diseases, given its multiple therapeutic benefits [275].

In a study using C57BL/6J mice treated to Ang II infusion and saline, systolic blood pressure was measured with a tail cuff system [276]. Histopathological staining was used to assess vascular remodeling and oxidative stress, while vasodilatory function was evaluated in the aortic ring. The findings revealed that administering GA significantly lowered vascular inflammation, fibrosis, and Ang II-induced hypertension. Additionally, GA improved vascular endothelial function and lowered oxidative stress in aortas infused with Ang II. Functionally, the treatment with GA diminished the Ang II-induced upregulation of the immunoproteasome catalytic subunits β2i and β5i, leading to a decrease in the proteasome’s trypsin-like and chymotrypsin-like activities. This suppression prevented the degradation of eNOS and maintained NO levels. Furthermore, the beneficial effects of GA were markedly diminished by blocking eNOS activity with the specific inhibitor L-NG-nitroarginine methyl ester. This study highlights GA as a unique immunoproteasome inhibitor, suggesting its potential as a therapeutic agent for hypertension and vascular dysfunction [276].

## 5. Food Applications

### 5.1. Active Packaging Systems

In recent years, active packaging systems have become a promising innovation in food preservation, addressing the increasing consumer demand for biodegradable, biocompatible, and environmentally friendly packaging materials. Moreover, these systems can effectively prolong the shelf life of food products due to the presence of a wide range of active compounds in their structure, including antioxidants, antimicrobials, and nutraceuticals [277,278,279]. In this respect, the incorporation of phenolic acids, e.g., GA (GA), can provide an additional protection layer against external spoilage factors, e.g., moisture, oxygen, and microorganisms, in active films and coatings [280]. Therefore, this strategy helps in reducing spoilage, extending shelf life, and maintaining the safety and quality of food products during storage, not only as a physical barrier but also by delaying the deterioration of the food’s nutritional and sensory properties.

#### 5.1.1. Fish and Seafood Products

The consumption of fish and seafood products has increased due to their high nutritional value, including polyunsaturated fatty acids (PUFAs), essential minerals, and easily digestible proteins. These bioactive compounds are also favorable for the growth of foodborne pathogens and lipid oxidative reactions, making marine-based products highly susceptible to quality deterioration (Table 6) [281]. To overcome these issues, various GA-loaded films and coatings have been developed to enhance the storage quality of fish fillets stored at 4 °C (Table 5). For instance, [282] showed that pectin (PE)-GA coatings could significantly reduce the total viable count (TVC) of fresh Japanese sea bass fillets more effectively than PE or GA treatments alone during storage at 4 °C for 20 days. Moreover, fish fillets treated with a PE-GA coating showed lower levels of lipid oxidation, total volatile basic nitrogen (TVB-N), and total sulfhydryl groups, while maintaining a higher Ca^2+^-ATPase activity compared to untreated, PE-coated, and GA-treated fillets. In another study by the authors of [283], chitosan (CS) was grafted onto GA and protocatechuic acid (PA), using a carbodiimide coupling method to create copolymers, namely, CS-g-GA and CS-g-PA. The CS-g-GA copolymer exhibited a higher grafting rate (110.82 mg GA/g) compared to CS-g-PA (62.63 mg PA/g). Both copolymers improved their rheological properties, thermal stability, and antioxidant activity over pure CS. Moreover, when CS-g-GA and CS-g-PA were applied to refrigerated sea bass fillets, these coatings significantly inhibited microbial growth and delayed the deterioration of texture, color, and sensory quality. Overall, these findings indicate the potential of GA-loaded coatings for enhancing the shelf life and quality of Japanese sea bass fillets as promising food-grade preservatives. In further studies, Wong et al. [284] and Song et al. [285] focused on the development of novel GA-containing films for the preservation of tilapia fillets. For this purpose, one study utilized a polyethylene (PE) film coated with CS and GA using plasma modification technology. This process effectively bonded CS and GA to the PE surface, resulting in a coating (GA/CS/PE) with superior antioxidant properties compared to the individual CS/PE and GA/PE films. The GA/CS/PE film significantly inhibited microbial growth, reducing TVC by 1.52 log CFU/g, and delayed TVB-N production below 15 mg/100 g after 14 days of storage. Additionally, the GA/CS/PE and GA/PE films exhibited better thiobarbituric acid inhibitory effects, showing lower malondialdehyde (MDA) levels (0.24 mg/kg and 0.26 mg/kg, respectively) compared to the control (0.30 mg/kg) on day 14 [284]. Another study aimed to develop collagen (Col) and zein (ZN) electrospun films encapsulated with different concentrations of GA to preserve tilapia fillets. The obtained Col/ZN/GA films were smooth and bead-free, with an average diameter of 272–384 nm, and GA was well-distributed in the protein matrix through hydrogen bonds. The results showed that the incorporation of GA reduced the hydrophobicity of the films, while significantly increasing their antioxidant activity. The preservation tests also revealed that Col/ZN/GA films containing 8% GA (GA8) effectively delayed the quality deterioration of tilapia fillets and extended their shelf life by at least two days. Thus, GA-loaded electrospun films, particularly GA8, show great potential as active packaging materials to extend the shelf life of fresh fish, offering promising applications in the fish-filleting industry [285].

In addition to Japanese sea bass and tilapia, the potential applications of GA-loaded coatings and films in maintaining the quality and extending the shelf life of other fish fillets have been investigated. In this regard, Wu et al. [286] found that combining GA and CS significantly enhanced the quality and shelf life of Pacific mackerel (*Pneumatophorus japonicus*) fillets during cold storage. The authors demonstrated that the CS-GA coating was more effective than both treatments alone in inhibiting biogenic amine formation, protein decomposition, microbial growth, lipid oxidation, and nucleotide breakdown, while also maintaining superior sensory properties. This treatment also extended the shelf life of mackerel by 6 days compared to the control group. Similarly, Zarandona et al. [287] evaluated the effects of active coatings containing GA and CS on the quality of Atlantic horse mackerel (*Trachurus trachurus*) fillets over 13 days of cold storage. All CS-based coatings significantly reduced microbial growth by more than two log cycles, with those containing CS nanoparticles proving most effective, possibly due to increased contact surface with the fish fillets. These nanoparticle coatings also resulted in the lowest TVB-N and maintained pH values below 7, indicating better preservation properties. In addition, these coatings showed the lowest thiobarbituric acid reactive substance (TBARS) values, attributed to the sustained release of GA, which prevented lipid oxidation and preserved the texture and color of the fish fillets during storage. In another study, Li et al. [288] developed zein/gelatin (ZG) composite nanofibers loaded with ε-polylysine (PL) and GA for the preservation of tuna fish (*Thunnus albacares*) pallets. The PL/GA/ZG films exhibited a well-distributed fiber morphology, with an average diameter of 810 ± 150 nm. The results also indicated that the addition of GA improved the thermal stability and antioxidant properties of the films, while PL enhanced their antibacterial effectiveness against *Shewanella putrefaciens*. Moreover, the composite films demonstrated significant benefits in preserving tuna fillets at 4 °C, showing reduced TVC and TVB-N, minimized fat oxidation, and delayed texture deterioration, thus extending their shelf life by three days compared to the control. This study highlights that GA, when combined with PL in food packaging systems, enhances both antioxidant and antimicrobial properties, offering a promising approach for extending the shelf life and maintaining the quality of seafood products. In a further study, Xiong et al. [289] developed an edible coating using gelatin (GE) extracted from salmon bones, combined with CS, GA, and clove oil (CO) for preserving fresh salmon fillets during cold storage (4 °C) for 15 days. The results indicated that the GE-CS-GA-CO coating could improve the quality of the fillets most effectively, in terms of pH, color, lipid and protein oxidation, and microbial growth, as well as antioxidant and antimicrobial activities, extending the shelf life of salmon fillets by at least 5 days. These findings are consistent with a study by Fu et al. [31], who developed an antioxidant and antimicrobial agarose-based coating grafted with GA through the carbodiimide coupling method. The structural characterization confirmed the successful grafting of GA onto agarose, achieving a grafting ratio of up to 13.73%. This process markedly enhanced the antioxidant and antimicrobial activities of the agarose, with increased 1,1-Diphenyl-2-picrylhydrazyl (DPPH) radical scavenging activity, and an up to 100% inhibition of *E. coli* and *S. aureus*. The preservation tests also showed that the GA-g-agarose coating effectively prevented fat oxidation, inhibited bacterial growth, reduced moisture loss, blocked light, and improved the overall quality of grass carp (*Ctenopharyngodon idellus*) fillets during refrigeration.

In summary, the use of GA-loaded coatings and films has shown significant potential in preserving the quality and extending the shelf life of various fish fillets, such as Pacific mackerel, Atlantic horse mackerel, tuna, salmon, and grass carp. These studies further highlight the effectiveness of GA, especially when combined with other bioactive compounds, in inhibiting microbial growth, reducing lipid oxidation, and maintaining the sensory properties of fish fillets during storage, offering promising solutions for seafood preservation.

**Table 6:** Recent applications of GA in active packaging systems for food preservation.

Food Products	Film/Coating Matrix	GA Concentration	Other Active Compounds	Storage Conditions	Highlights	References
**Fish and seafood products**						
Japanese sea bass (*Lateolabrax japonicus*) fillets	Pectin	5% (*w*/*v*)	-	20 days, 4 °C	□ Lower TVB-N, lipid oxidation, and total sulfhydryl groups □ The coated samples showed the highest sensory quality rating	[282]
	CS	15 mM	PA	10 days, 4 °C	□ CS-grafted GA showed a higher GR (110.82 mg GA/g) than CS-grafted PA (62.63 mg PA/g) □ Higher thermal, rheological, and antioxidant properties than pure CS □ Delayed the deterioration of texture, color, and sensory quality	[283]
Tilapia (*Orechromis niloticus*) fillets	PE + CS	1% (*w*/*w*)	-	14 days, 4 °C	□ Higher antioxidant and antimicrobial activities □ Inhibited TVC and TVB-N □ Lower TBARS value on day 14	[284]
	Collagen + zein	1–10% (*w*/*w*)	-	10 days, 4 °C	□ The electrospun fibers exhibited a smooth nanostructure with no beads □ GA formed hydrogen bonds with the protein matrix □ Prolonged the shelf life of the fillets for at least two days, especially at a concentration of 8%	[285]
Pacific mackerel (*Pneumatophorus japonicus*) fillets	CS	5% (*w*/*v*)	-	12 days, 4 °C	□ Inhibited protein decomposition, nucleotide breakdown, microbial growth, and lipid oxidation up to 6 days □ Delayed the deterioration of sensory quality	[286]
Horse mackerel (*Trachurus trachurus*) fillets		10 wt%	-	14 days, 4 °C	□ Decreased microbial growth in more than two log cycles □ Lower TVB-N and TBARS values	[287]
Yellowfin tuna (*Thunnus albacares*) fillets	Zein + gelatin	1 g	PL	15 days, 4 °C	□ Higher average diameter with well-distributed morphology □ Improved thermal, antioxidant, and antimicrobial properties □ The combined films effectively inhibited TVC, TVB-N, lipid oxidation, and texture deterioration up to 3 days	[288]
Atlantic salmon (*Salmo salar*) fillets	Gelatin + CS	0.2% (*w*/*v*)	CO	15 days, 4 °C	□ Higher antioxidant and antimicrobial activities □ The combined coatings prolonged the shelf life of the fillets up to 5 days	[289]
Grass carp (*Ctenopharyngodon idellus*) fillets	Agarose	0.0350–0.1373 g	-	15 days, 4 °C	□ GA was grafted onto the C_6_-OH of D-galactose in agarose, with a highest GR of 13.73% □ Higher antioxidant and antimicrobial activities □ Lower viscosity, gel strength, and gelling temperature □ Inhibited microbial growth and lipid oxidation	[31]
**Meat products**						
Pork	CS	5 mL	-	18 days, 4 °C	□ Higher antioxidant and antimicrobial activities □ Lower TVB-N and TBARS values □ Prolonged the shelf life of the pork meat from 6 to 18 days	[290]
		0.2 and 0.4% (*w*/*w*)	-	20 days, 4 °C	□ Higher antioxidant activity □ Lower lipid oxidation and myoglobin oxidation □ Improved the safety and quality of samples in MAP environment	[291]
	Collagen + CS	NS	PL	15 days, 4 °C	□ Improved structural and UV barrier properties □ Higher antioxidant and antimicrobial activities □ Prolonged the shelf life of the pork meat by approximately 5 days	[292]
	CYS + CS	40 g	-	One day, 25 °C	□ Better light transmittance and thinner thickness □ Improved the tensile strength □ Improved the quality of pork during storage compared to PE film packaging	[293]
Beef	CS	0.1 and 0.3% (*w*/*v*)	-	21 days, 4 °C	□ Reduced spoilage bacteria count, TVB-N, and TBARS □ Delayed lipid oxidation and color deterioration	[294]
	Gelatin + CS	0.5%	CGA and RES	12 days, 4 °C	□ Lower TVC, TVB-N, and TBARS values □ The combined coatings prolonged the shelf life of beef at least 3–6 days	[295]
	CA	NS	ZIF-8	12 days, 4 °C	□ Improved mechanical strength and UV barrier properties □ Reduced WVP, MC, and SR □ Higher antioxidant and antimicrobial activities	[296]
**Fruits and vegetables**						
Strawberry	CS	15 mM	-	14 days, 4 °C, under UV-A light	□ Higher reduction of *E. coli* compared to the control after 180 min exposure to UV-A □ The photo-irradiated coatings did not significantly affect the mold decay incidence in strawberries □ The firmness value did not show significant differences during storage	[297]
Banana	mPLA	0.0301 g	-	14 days, 25 °C	□ Improved mechanical and antioxidant properties □ Retained the firmness and green peel color of bananas after storing for 14 days	[298]
Mango	CS or CG	0.075 or 0.15% (*w*/*v*)	-	14 days, 20 °C, 60–70% RH	□ Lower pH, TSS, and TSS/acid ratio □ Higher antioxidant activity (lower IC50 values) after one week of storage □ Delayed ripening during two weeks of storage	[299]
Cherry tomato	CS	GA/CS ratio of 1:3 (*w*/*w*)	-	10 days, 15 °C	□ Improved antioxidant activities in scavenging hydroxyl, DPPH, and superoxide anion radicals □ Protected the ascorbate–glutathione cycle of cherry tomatoes □ Inhibited enzymatic browning	[300]
	PLA-PBAT	1, 5, and 10 wt%	TA	20 days, 25 °C	□ Improved tensile strength and UV barrier properties □ High antimicrobial activity against *E. coli* and *L. monocytogenes*, especially for those containing 10 wt% GA □ Enhanced the shelf life of cherry tomatoes for up to 20 days of storage at room temperature	[301]
Green chili	CS + pullulan	5, 10, and 15 wt%	-	18 days, 25 °C	□ Improved tensile strength, WVP, and oxygen and UV barrier properties, especially for those containing 15 wt% GA □ Lower overall migration than the acceptable limit of 10 mg dm^−2^ □ Higher antioxidant and antimicrobial activities	[302]

#### 5.1.2. Meat Products

Meat, a nutritious food rich in essential proteins, lipids, and micronutrients, is highly susceptible to quality deterioration due to its nutritional composition and high moisture content, leading to significant losses along the supply chain [303]. Traditional packaging, while providing necessary protection and extending shelf life by limiting microbial growth and oxidation, poses environmental challenges due to its difficulty in recycling [304]. To address these issues, researchers are developing eco-friendly, biodegradable, and even edible packaging materials from renewable resources [305]. Recent advancements in this field have focused on using GA-loaded films and coatings, which have demonstrated a high effectiveness in preserving meat quality by enhancing their antioxidant and antimicrobial properties, thus extending the shelf life of meat products, while offering sustainable packaging solutions (Table 6).

Zheng et al. [290] prepared GA-g-CS coatings using recombinant bacterial laccase from *Bacillus vallismortis* fmb-103 to enhance the shelf life of pork loin meat during storage. A structural analysis through Fourier-transform infrared (FT-IR) and ultraviolet–visible (UV-VIS) spectroscopy confirmed a successful grafting between GA and CS. Moreover, the GA-g-CS exhibited significantly improved antibacterial activity against various pathogens, e.g., *E. coli*, *Salmonella*, *L. monocytogenes*, *Acinetobacter*, *Pseudomonas*, *S. aureus*, and *B. thermosphacta*, and increased antioxidant properties, as demonstrated by the DPPH radical scavenging results. The GA-g-CS coating could also retard the growth of spoilage bacteria, reduce TVB-N and TBARS values, and extend the meat’s shelf life from 6 to 18 days at 4 °C. In another study, Fang et al. [291] investigated the effectiveness of edible coatings based on GA and CS in preserving fresh pork quality in modified atmosphere packaging (MAP) at 4 °C. Pork loins were coated with 2% CS, 0.2% GA in 2% CS, or 0.4% GA in 2% CS. The results indicated that the antimicrobial activity of the CS coating improved with the addition of GA, while significantly reducing the lipid and myoglobin oxidation of the meat. However, the 0.4% GA coating showed a pro-protein oxidation effect, highlighting the need for an optimal GA concentration. In a further study, Zheng et al. [292] developed active packaging films composed of Col, GA-functionalized CS, and PL to extend the shelf life of pork stored at 4 °C. The FT-IR analysis confirmed hydrogen bonding between Col and GA, while scanning electron microscopy (SEM) images revealed a compact film structure with some aggregation at higher PL levels. These films demonstrated excellent light barrier properties, with a UV light transmittance below 4%, and significantly enhanced antioxidant activity due to the presence of GA and PL, increasing the DPPH free radical scavenging rate by 84.35%. Additionally, the film containing 5 wt% PL exhibited strong antimicrobial activity against *S. aureus*, good tensile strength, and effectively reduced lipid oxidation and bacterial growth in pork, extending its shelf life by approximately 5 days compared to PE film. Moreover, Rong et al. [293] focused on developing edible films based on GA-induced Chinese yam starch (CYS) and CS for pork preservation. The GA/CYS/CS films showed better light transmittance and thinner thickness. Besides, the SEM images and mechanical tests showed good compatibility between the GA, CYS, and CS, with enhanced tensile strength due to hydrogen bonds between CS and CYS. The sensory analysis also indicated that GA/CYS/CS films improved pork quality during storage, even better than PE film. These films offer excellent mechanical, antibacterial, and oxidation-resistant properties, showcasing their potential for practical applications in pork preservation and their role in mitigating environmental pollution.

Similar to pork meat, beef is also highly susceptible to oxidation due to the presence of high levels of nutritional compounds, e.g., PUFAs, leading to quality deterioration and economic losses. To address this issue, a study assessed the effectiveness of CS-based coatings, with/without GA, in preserving Jeju black cattle (JBC) beef during refrigerated storage. Fresh beef steaks were coated with 2% CS, 2% CS with 0.1% GA, or 2% CS with 0.3% GA, then stored at 4 °C. The results showed that, while CS alone reduced spoilage bacteria, TVB-N, and lipid oxidation compared to the control, the addition of GA significantly enhanced these protective effects. The combined CS-GA coatings were more effective in inhibiting bacterial growth, oxidation, and discoloration over 21 days, suggesting that CS and GA coatings are promising bio-preservatives for the meat industry [294]. In another study, Zou et al. [295] investigated the effects of incorporating chlorogenic acid (CGA), resveratrol (RES), and GA into a gelatin–chitosan (GE-CS) edible coating on fresh beef preservation. The results revealed that while CGA provided the most significant improvement in preserving beef color and delaying the increase in TVB-N, GA also demonstrated notable preservation benefits. Specifically, beef coated with GE-CS-GA had the lowest levels of TBARS (0.76 mg MDA/kg) and TVC (6.0 log cfu/g) on the 12th day of storage, indicating reduced lipid oxidation and microbial growth. Overall, the incorporation of polyphenols into the coating extended the beef shelf life by approximately 3–6 days compared to the control. Furthermore, Zhang et al. [296] developed a carrageenan (CA) matrix incorporated with a zeolite imidazolium skeleton-8 (ZIF-8) for the delivery of GA, resulting in CA-GA@ZIF-8 films. These films demonstrated enhanced mechanical strength and UV protection, as well as a reduced moisture content, water vapor permeability, and swelling rate. They also showed a sustainable GA release, controlled Zn^2+^ migration, high antioxidant (i.e., 83.29% DPPH and 62.11% 2,2′-azino-bis(3-ethylbenzothiazoline-6-sulfonic acid) (ABTS) radical scavenging, and antimicrobial (i.e., 99.51% against *E. coli* O157:H7) activities. Moreover, the preservation experiments indicated that CA-GA@ZIF-8 films effectively maintained beef freshness, reduced microbial growth, and inhibited lipid oxidation, highlighting their potential for improving meat quality preservation.

Overall, these studies demonstrate that GA-loaded coatings and films can effectively enhance the safety and quality of pork and beef meat products during storage, emphasizing their potential as a sustainable food preservation method. However, further research is needed to investigate the optimal concentration of GA in these food products under different environmental conditions.

#### 5.1.3. Fruits and Vegetables

Fruits and vegetables are classified as highly perishable food products, with a short post-harvest life, which results in significant physiological and biochemical changes during storage. This leads to a substantial global waste of 500 million tons per year, with about 20–30% of post-harvest fruits and vegetables lost, due to microbial growth, ethylene-induced rapid senescence, oxidative browning, and other spoilage issues. This waste not only impacts global food security but also contributes to environmental problems, such as excessive carbon emissions and waste disposal [306].

Recent studies have suggested that food packaging, particularly that containing active ingredients, is a widely adopted and cost-effective solution to these issues, which provides protective barriers against external contamination, thereby maintaining the quality and extending the shelf life of fruits and vegetables during storage (Table 5). For instance, strawberries are generally susceptible to contamination by pathogens and mold during post-harvest transportation and storage. Therefore, Zhang et al. [297] explored the potential use of a CS-GA coating combined with 360 nm UV-A light to enhance the antimicrobial properties and shelf life of strawberries during cold storage. The CS-GA coating allowed an 80% UV-A transmission, while blocking most UV-C (200–280 nm) light, effectively reducing *E. coli* O157:H7 by 2.3 log after 180 min of UV-A exposure. This reduction was significantly higher than those treatments without UV-A. Moreover, the treatment did not significantly affect the mold incidence or firmness of the strawberries, though it initially reduced their redness and yellowness. These findings suggest that photo-irradiated CS-GA coatings can effectively improve the microbial safety of strawberries, without compromising their quality. In another study, Taechutrakul et al. [298] prepared different concentrations (1, 2, and 4 wt%) of multibranched polylactic acid-GA (mPLA-GA) through ring-opening polymerization and then combined them with PLA to form PLA/mPLA-GA film strips. These strips showed improved antioxidant activity, mechanical properties, and oxygen scavenging capabilities compared to PLA and PLA/GA strips alone. In addition, the PLA/mPLA-GA strips significantly delayed banana ripening, maintaining peel color and firmness over 14 days at room temperature. In a further study, Awad et al. [299] evaluated the effects of CS, GA, and chitosan gallate (CG) on the biochemical changes and post-harvest quality of ‘Hindi-Besennara’ mangoes during two weeks of storage at 20 ± 2 °C. The results indicated that both GA and CG treatments could effectively reduce the weight loss and preserve the acidity and vitamin C levels of mangoes, while lowering their pH and total soluble solids (TSSs) compared to the control. Besides, GA-treated fruits exhibited a higher antioxidant capacity and lower total phenols after one week, while all treatments decreased α-amylase activity and increased peroxidase activity, thus delaying ripening and maintaining fruit quality. GA-loaded packaging materials can also effectively prolong the shelf life of cherry tomatoes and enhance their quality during storage, as evidenced by Zhang et al. [300] and Sharma et al. [301]. In this respect, the authors demonstrated that the GA-g-CS coating exhibited a high free radical scavenging ability that could effectively protect the ascorbate–glutathione cycle and antioxidant enzyme system in cherry tomatoes, inhibiting enzymatic browning and delaying post-harvest senescence [300]. Similarly, the incorporation of GA and tannic acid (TA) into a biodegradable Poly Lactide-Poly (Butylene Adipate-Co-Terephthalate) (PLA-PBAT) polymer resulted in films with enhanced UV barrier properties, tensile strength, and antimicrobial activity. These TA and GA composite films also maintained the quality of cherry tomatoes for up to 20 days at room temperature, showcasing their potential as active packaging solutions for extending the shelf life of perishable products [301]. Furthermore, Gasti et al. [302] developed biofilms based on CS and pullulan (PL), functionalized with various concentrations of GA, i.e., 5, 10, and 15 wt%, using the solvent casting technique. The CS/PL/GA films, particularly those with 15 wt% GA, showed a high tensile strength, excellent UV blocking, and significantly enhanced water vapor and oxygen barrier properties. These films also exhibited strong antioxidant (∼42.54%) and antimicrobial activities against foodborne pathogens, e.g., *E. coli*, *S. aureus*, and *A. niger*. Moreover, the preservation tests showed that CS/PL/GA films effectively extended the shelf life of green chilies by up to 18 days at room temperature without compromising quality, suggesting that these films are highly promising for active packaging applications in fresh food preservation.

### 5.2. Functional Foods

The concept of functional foods, introduced in Japan in the 1980s, refers to foods enriched with compounds that offer additional physiological benefits, such as reducing disease risk or treating illnesses. Defined by the Functional Food Center (FFC) in 2014, these foods contain bioactive compounds that provide clinically proven health benefits [307]. In this regard, GA can play a crucial role in the production of functional food products, particularly due to its excellent antioxidant and antimicrobial activities. Previous studies revealed that GA can be successfully incorporated into various foods and beverages, such as bakery, dairy, and meat products, enhancing their health benefits. However, this may also affect the techno-functional and sensory attributes of the final product, which should be optimized in novel functional food formulations, addressing the consumer demand for safe, natural, nutritious, and tasty additives.

The Maillard reaction (MR) is pivotal in enhancing the sensory qualities of bakery products, contributing to their aroma, flavor, and color. However, it also leads to the formation of harmful by-products, such as advanced glycation end products (AGEs) and acrylamide, which are associated with various health risks, including cancer and chronic diseases. Given the widespread consumption of bakery products, identifying strategies to mitigate these harmful by-products is crucial for public health. In this respect, various methods have been developed to inhibit the MR in food processing, including the use of enzymes andinnovative technologies, e.g., high-pressure processing and ohmic heating, and chemical modifications [308]. On the other hand, recent studies have highlighted the promising efficacy of phenolic compounds, e.g., GA, in reducing the formation of harmful MR by-products in bakery products. For instance, the addition of only 0.1% of phenolic compounds to a bread formulation significantly reduced its acrylamide levels by 16.2 to 95.2%, with GA showing a strong inhibitory effect [309]. Moreover, the addition of GA reduced 5-hydroxymethylfurfural (5-HMF) formation by 49% in glucose/arginine systems and by 54% in sucrose/arginine systems, while also enhancing antioxidant activity and browning, indicating a positive effect on food quality [143]. Furthermore, the inhibitory effect of GA on digestive enzymes and its ability to form V-type starch complexes leads to a lower predicted glycemic index (pGI) [310,311,312]. For instance, the GA-fortified buckwheat Wantuo (BWT) showed significant changes in starch digestibility, increasing the content of resistant starch to 78.48% with the addition of 5% GA, compared to 8.62% rapidly digested starch [313]. Similarly, in pea starch–GA complexes, the resistant starch content increased from 16.63 to 26.67% after high-pressure homogenization, and rapidly digestible starch decreased from 29.67 to 17.07%, which highlights the promising role of GA in enhancing the nutritional profile of bakery products [314]. In conclusion, GA can play a crucial role in the development of functional bakery products by reducing harmful MR by-products, enhancing antioxidant properties, and reducing starch digestibility. The incorporation of GA into bakery formulations not only mitigates the health risks associated with harmful compounds but also enhances the overall quality and shelf life of the products, making GA an essential ingredient in the food industry.

GA has also demonstrated considerable potential in enhancing the quality and health benefits of dairy products, as evidenced by various studies. When GA was added to infant milk substitutes, it effectively prevented the oxidation of unsaturated fatty acids under oxidative stress conditions induced by UV-C radiation. Specifically, the addition of GA at concentrations up to 3 mM resulted in a significant reduction in malondialdehyde (MDA) levels, a marker of lipid peroxidation [315]. Moreover, in fish oil-enriched cow milk (CM) and soymilk (SM) emulsions stored at 4 °C, GA and its derivatives, particularly octyl gallate and propyl gallate, demonstrated significant antioxidant effects. These gallates were more effective due to their higher interfacial activity at the oil–water interface. For instance, octyl gallate showed the highest antioxidant effect, leading to a substantial reduction in the peroxide values and volatile compounds associated with lipid oxidation. This highlights the importance of the molecular structure of GA derivatives in enhancing the oxidative stability of dairy emulsions [316]. GA also plays a crucial role in improving the gelation properties of milk. When TA (0.1–1% *w*/*w*) and GA (0.3–1% *w*/*w*) were added to skim milk before acidification, the resulting gels exhibited faster gelation times and a higher storage modulus (G′). Specifically, the addition of up to 0.8% GA resulted in a significant decrease in water mobility (T2 time) without affecting the syneresis index (SI). However, at 1% GA, there was a notable decrease in G′ and an increase in SI, indicating optimal concentrations for maintaining gel strength and stability. Moreover, lowering the temperature from 30 to 5 °C further increased G′, due to enhanced hydrogen bonding in the presence of GA [317]. Furthermore, GA can effectively improve the shelf life and stability of dairy products during storage, e.g., milk and ice cream. In milk fortified with chitooligosaccharide–GA conjugate (CGC), the microbial counts and lipid oxidation markers were significantly lower compared to control samples. For example, CGC-fortified milk with 2.50% CGC had the lowest microbial counts and oxidative markers, extending the shelf life at 4 °C to a minimum of 6 days compared to less than 4 days for the control [318]. In ice cream production, the combination of GA with transglutaminase also resulted in superior physicochemical properties, including a higher melting time and stability, and overall sensory acceptance (91%). Notably, the ice cream mixture showed a decrease in pH and an increase in titratable acidity after aging, demonstrating improved structural and sensory characteristics [319].

In a study on irradiated turkey sausages, GA at a concentration of 0.02% effectively improved the redness (a*) values, i.e., 1.49 (0 kGy), 2.03 (1.5 kGy), and 2.29 (3.0 kGy), showcasing its impact on maintaining color quality after irradiation. Additionally, GA significantly decreased the total volatiles, although it had a minimal effect on the off-flavor caused by irradiation. This study illustrated the capability of GA in enhancing the sensory attributes and oxidative stability of meat products subjected to irradiation [320]. Furthermore, López-Romero et al. [321] reported that the combined application of GA and eugenol could significantly enhance the thermal inactivation of *Salmonella* spp. in ground chicken, highlighting their synergistic effect on food safety during thermal processing. GA also exhibited potent antimicrobial properties against other food pathogens, as demonstrated by its effect on *Yersinia enterocolitica* in pork. At a concentration of 5 mg/g, GA reduced the bacterial count by 2 log cycles during 3 days of storage at 4 °C. This reduction was attributed to GA-induced membrane damage in *Y. enterocolitica*, evidenced by a significant decrease in intracellular ATP and pH levels [114]. In another study, Anvar et al. [322] revealed that the encapsulation of GA in nanoliposomes (N-GA) further enhanced its antimicrobial efficiency in minced meat. The minimum inhibitory concentration (MIC) of N-GA for *S. aureus* was 0.62 ± 0.02 mg/mL, while the MIC for *E. coli* was 1.25 ± 0.1 mg/mL. After 18 days of storage, the pH and TVB-N values were lowest in the N-GA 2% treatment (i.e., pH = 6.5; TVB-N = 27 mg N/100 g), indicating better preservation and higher sensory acceptance compared to the other treatments. Therefore, these findings underscore the significant role of GA and its derivatives in enhancing the sensory attributes, oxidative stability, and microbial safety of various meat products during storage. On the other hand, the fortification of food products with phenolic compounds, such as GA, may also offer multiple health benefits, including anticancer, antidiabetic, anti-inflammatory, and neuroprotective effects. However, the complexity of food matrices poses challenges in the accurate analysis of polyphenols, due to their interactions with carbohydrates, proteins, and lipids [323]. Despite these challenges, the use of GA in functional food matrices, particularly in bakery products, demonstrates a promising approach to enhancing the health profile of these products, while managing the complexities of polyphenol interactions and stability during processing.

## 6. Disadvantages of GA in Human Health and Food Products

Despite several beneficial effects, GA also presents several disadvantages, particularly when used in food products and concerning its impact on human health. This section explores the potential adverse effects of GA and the limitations associated with its application in food industries.

### 6.1. Toxicity

While GA is recognized for its antioxidant properties, it can exhibit cytotoxic and genotoxic effects under certain conditions. High concentrations of GA have been shown to induce oxidative stress, leading to cellular damage. Studies have demonstrated that elevated levels of GA can cause DNA damage, potentially leading to mutagenesis or carcinogenesis. In vitro experiments using human cell lines, such as hepatocytes and lymphocytes, have revealed that gallic acid can induce apoptosis (programmed cell death) and necrosis. While this might be beneficial for targeting cancer cells, these effects can extend to normal cells when administered inappropriately high doses, raising concerns about its safety in both therapeutic and food applications [324,325,326]. While gallic acid is generally considered safe at typical dietary levels, excessive intake may lead to adverse effects.

### 6.2. Interaction with Drugs and Nutrients

Gallic acid may interfere with the metabolism of certain drugs and nutrients, leading to adverse interactions. For example, GA can inhibit enzymes such as cytochrome P450, essential for metabolizing various pharmaceuticals. This inhibition can result in altered drug efficacy or increased toxicity, posing risks for patients undergoing pharmacological treatment [141]. Additionally, GA’s ability to chelate metal ions could impact the bioavailability of essential nutrients like iron, potentially leading to nutrient deficiencies if consumed in excessive amounts [141].

### 6.3. Sensory Impact in Food Products

GA in food products can result in undesirable sensory changes. Known for its astringent and bitter taste, gallic acid can negatively affect the flavor profile of foods and beverages. For instance, its introduction into products like juices, wines, or teas can result in a more pronounced bitterness, potentially reducing consumer acceptance. This sensory impact limits the use of gallic acid in food formulations where taste and overall consumer satisfaction are critical [327,328]. The strong antioxidant activity of gallic acid can interfere with the natural oxidation processes that are essential for the development of flavor, color, and aroma in certain foods. This interference can lead to a reduction in the organoleptic qualities of foods, such as desirable browning reactions [329].

## 7. Conclusions and Future Perspectives

GA has garnered significant attention for its extensive therapeutic and functional applications, demonstrating a wide range of biological activities that contribute to human health. This literature review explored the chemical structure, resources, identification techniques, biological properties, and food applications of GA, underscoring its potential as a vital bioactive compound in both medicinal and nutritional contexts. GA is a trihydroxybenzoic acid found in various natural sources, which can be identified and qualified using various advanced analytical methods such as spectroscopy, chromatography (e.g., TLC, GC, HPLC), and capillary electrophoresis, ensuring the accuracy of research and applications in different fields. Generally, GA is known for its numerous biological activities. For instance, it has shown effectiveness against various bacterial strains, contributing to its potential use in treating infections. Moroever, its strong free radical scavenging ability makes GA a powerful antioxidant, protecting cells from oxidative stress. Additionally, GA reduces inflammation by modulating inflammatory pathways, which is beneficial in treating chronic inflammatory diseases. It also exhibits significant activity against fungal pathogens and viruses, enhancing its therapeutic scope. Furthermore, the potential anticancer, anti-allergic, anti-AD, anti-obesity, anti-diabetes, anti-hypertensive, and anti-asthmatic activities of GA and its derivates highlight the important role of these bioactive compounds in managing and preventing a wide range of health conditions. Therefore, the incorporation of GA into food products can offer substantial nutritional and techno-functional benefits. In this regard, previous studies have indicated that GA-loaded films and coatings significantly enhance the shelf life of food products during storage, mainly by providing high antioxidant and antimicrobial activities. Moreover, fortifying foods with GA may improve the oxidative stability, sensory attributes, and microbial safety of the final products, in addition to reducing harmful Maillard reaction (MR) by-products, making GA as an essential ingredient in various sectors of the food industry, especially bakery products.

The future of GA research and applications lies in overcoming the current challenges and exploring new opportunities for its utilization. In this respect, the development of novel encapsulation and delivery systems will be crucial to protecting GA during food processing and ensuring its effective release and absorption in the human body. In addition, investigating the synergistic interactions between GA and other bioactive compounds could enhance its efficacy and broaden its application scope. Therefore, the combination of GA with other phenolics, vitamins, or minerals could lead to novel functional foods with superior health benefits. Moreover, while the current applications of GA focus primarily on bakery, dairy, and meat products, future research should aim to incorporate GA into a wider range of food products, including juices, snacks, and ready-to-eat meals. This expansion could provide more accessible and convenient ways for consumers to benefit from the health-promoting properties of GA. On the other hand, conducting extensive clinical trials is essential to validate the therapeutic potential of GA observed in preclinical studies. These studies will help establish effective dosages, safety profiles, and dietary recommendations, facilitating the integration of GA into standard medical and nutritional practices.

In conclusion, GA holds enormous potential to enhance the health and well-being of consumers through its diverse biological activities and functional applications in various food products. However, continued research and innovation are essential to fully explore its functional benefits, overcome the existing challenges, and pave the way for GA to become a staple in therapeutic and nutritional interventions.

## Figures and Tables

**Table 1:** Overview of methods of determination and quantification of GA in different matrices.

Sample/Matrix Type	Sample Pretreatment	Sample Preparation	Analytical Method	Analytical Conditions	GA Concentration	References
Longan seed (*Dimocarpus longan* Lour.) and mango kernel (*Mangifera indica* L.) (Singapore)	Longan seed and mango kernel: freeze-dried (FD) (−50 °C, 24 h) Mango kernel: dried at 160 °C, then ground and stored at −20 °C	Longan seed: (1) FD/EtOH extraction al 70 °C, 1 h Mango kernel (2) FD/EtOH extraction at 70 °C, 1 h (3) FD/MeOH extraction at 70 °C, 1 h (4) FD/MeOH and hydrolysis at 35 °C, 16 h (5) FD/MeOH and hydrolysis at 85 °C, 2 h (6) 160 °C-dried/MeOH extraction at 70 °C, 1 h (7) 160 °C-heated/MeOH extraction and hydrolysis at 35 °C, 16 h (8) 160 °C-heated/MeOH extraction and hydrolysis at 85 °C, 2 h	RP-HPLC/DAD	Column: Shim-Pack VP-ODS column (250 × 4.6 mm) with a guard column GCP-ODS (10 × 4.6 mm); MP: H_2_O–AA (97:3, *v*/*v*) (A) and MeOH (B) in gradient mode: 10% B for 10 min to 70% B in 40 min; Tª: 40 °C; FR: 0.9 mL/min; λ = 280 and 360 nm	[GA] (mg/100 g seeds) (1) 23.3 (2) 185 (3) 20.0 (4) 84.1 (5) 163 (6) 414 (7) 535 (8) 838	[67]
*Nigella sativa* seeds (Nagpur, India)	Seeds were dried in air, ground, sieved, weighed and stored in an airtight container at room temperature	*N. sativa* powder was dissolved in 10 mL of MeOH, filtered, and distilled under reduced pressure.	RP-HPLC/DAD	Column: C18 (MakeGrace) (250 × 4.6 mm, 5 µm); MP: ACN:H_2_O (60:40, *v*/*v*) adjusted to pH 3.00 with 0.05% PA was used in isocratic mode; Tª: RT; FR: 0.5 mL/min; λ = 210 nm	[GA]: 0.4736 µg/mL	[68]
Five commercial pomegranate juices (PJ) with different brands (Mahshahr, Iran)	Samples were stored at 4 °C	5 mL of each sample was centrifuged at 3000 rpm for 20 min. The supernatant was filtered and diluted to 50 mL with distilled water; 10 mL of the above solution was transferred into a volumetric flask, and after the addition of α–cyclodextrin, adjusting the pH to 3.0 and filling it to the mark with 10% EtOH:H_2_O, it was sonicated for 20 min.	RP-HPLC/DAD	Column: C18 (250 mm × 4.6 mm, 5 µm); MP: ACN/diluted with PA 25.0 × 10^−3^ mol/L (15:85, *v/v*) in isocratic mode; FR: 1.0 mL/min; λ = 240 nm	[GA] (mg/L) PJ1: 3.98 PJ2: 2.41 PJ3: 1.30 PJ4: 3.00 PJ5: 4.98	[69]
Grape juice (GJ) and wine (W) (Sub-Middle São Francisco Valley, Brazil)	Sorting and separation of GJ varieties (GJA, GJB, GJC, GJD, GJE) and W varieties: WF, WG, WH	GJ/W previously diluted 500 μL + 1000 μL in phase A.	RP-HPLC/DAD	Column: Zorbax Eclipse Plus RP-C18 (100 × 4.6 mm, 3.5 µm) with a guard column C18 (12.6 × 4.6 mm, 5 µm); MP: 0.1 M PA in H_2_O (pH 2.0) (A) and 0.5% PA in MeOH (B) in a gradient mode: 0–5 min: 5% B; 5–14 min: 23% B; 14–22 min: 26% B; 22–25 min: 80% B; Tª: 35 °C; FR: 0.8 mL/min; λ = 280 nm	[GA] (mg/mL) GJA: 4.5 ± 0.4 GJB: 3.6 ± 2.9 GJC: 16.7 ± 3.5 GJD: 7.6 ± 1.2 GJE: 6.5 ± 1.0 WF: 26.4 ± 1.1 WG: 24.7 ± 1.0 WH: 16.9 ± 2.6	[70]
Skins, pulps, seeds, canes, and leaves from grapes (*V. vinifera* L) (Urmia, Iran)	Sorting, separation, and crushing of different parts of grape varieties: Muscat Alexanderia (MA), Hosseini (Hos), Ghara Shira (GShi), Agh Shani (AG), Ghara Shani (GSha), and Ghara Ghandome (GG)	UAE: Powdered grape parts were weighed and mixed with 5 mL. MeOH:HCl (99:1, *v*/*v*) for ultrasonic extraction at 25 °C and a frequency of 35 kHz for 20 min. Then, samples were taken out and left at RT for 30 min. The extract was filtered and the remaining solids were extracted again under the same conditions.	RP-HPLC/DAD	Column: C18, (250 × 4.6 mm, 5 µm) and pre-column; MP: H_2_O:THF:TFA (98:2:0.1, *v*/*v*/*v*) (A) and MeOH:THF:TFA (98:2:0.1, *v*/*v*/*v*) (B) in a gradient mode: 17% B for 2 min: 17% B for 2 min increasing to 25% B after 5 min to 35% B after a further 8 min and to 50% B after a further 5 min; Tª: 25 °C; FR: 1 mL/min; λ = 278 nm	[GA] (μg/g) MA: skin: 122 ± 7, pulp: 109 ± 5; seed: 87 ± 3; cane: 118 ± 4; leaf: 1.4 ± 0.1; Hos: skin: 143 ± 6, pulp: 128 ± 7, seed: 87 ± 5, cane: 102 ± 4, leaf: 0.9 ± 0.1; GShi: skin: 238 ± 13, Pulp:153 ± 8, seed: 77 ± 4, cane: 132 ± 8, leaf: 1.3 ± 0.2; AG: skin: 220 ± 13, pulp: 178 ± 8, seed: 77 ± 4, cane: 141 ± 7, leaf: 1.9 ± 0.2; GSha: skin: 319 ± 17, pulp: 192 ± 10, seed: 91 ± 5, cane: 153 ± 8, leaf: 2.6 ± 0.2; GG: skin: 127 ± 7, pulp: 87 ± 4, seed: 67 ± 4, cane: 101 ± 6, leaf: 1.1 ± 0.1	[71]
Three different varieties of Camellia seed oils (China)	*C. sinensis* (*CS*)*:* (1) H. Xinyang; (2) J. Lushan; (3) S. Rizhao; (4) H. Enshi; (5) Z. Hangzhou; (6) F. Quanzhou *C. oleifera* (*CO*): (7) A. Huangshan; (8) J. Ganzhou; (9) G. Liuzhou; (10) H. Chenzhou; (11) H. Huaihua; (12) F. Shanming *C. chekiangoleosa* (*CC*)*:* (13) J. Dexing; (14) Z. Kaihua; (15) H. Loudi All kinds of Camellia seeds were dried, unshelled, and crushed to obtain oil. The oil samples were placed in glass bottles, and stored in the dark at RT until further extraction	Liquid–liquid extraction assisted by centrifugation.	HPLC-ESI-Q-TOF-MS	HPLC conditions: Column: SPURSIL-C18 (150 × 2.1 mm, 2.1 µm); MP: 0.1% AA in H_2_O (A) and 0.1% AA in ACN (B) in a gradient mode: 0–20 min, 0–50% B; 20–25 min, 50–100% B; Tª: 30 °C; FR: 0.4 mL/min. ESI conditions: negative ionization mode; capillary: 4000 V; nebulizer pressure: 30 psi; fragment voltage: 140 V; drying gas FR: 9 L/min; gas Tª: 190 °C; N_2_ sheath gas Tª: 350 °C; N_2_ sheath gas FR: 10 L/min; Scan range, *m*/*z:* 100–1000 in full scan mode; collision energy: 10–40 eV. Quantitative analysis was performed by HPLC-QqQ-MS with the following gradient elution: 0–30 min, 0–10% B; 30–50 min, 10–40% B; 50–55 min, 40–0% B.	[GA] (μg/g) CS1: 2.2114 ± 0.0069 CS2: 2.3097 ± 0.0239 CS3: 1.9689 ± 0.0113 CS4: 2.5764 ± 0.0099 CS5: 1.8805 ± 0.0019 CS6: 2.3964 ± 0.0201 CO7: 0.5150 ± 0.0011 CO8: 0.4640 ± 0.0051 CO9: 0.9720 ± 0.0102 CO10: 1.5580 ± 0.0014 CO11: 0.5169 ± 0.0051 CO12: 0.7012 ± 0.0009 CC13: 1.7910 ± 0.0062 CC14: 1.4881 ± 0.0098 CC15: 1.1243 ± 0.024	[72]
Two varieties of *Psidium guajava* L. (Motril, España)	*Pyrifera* (Pyr) and *pomifera* (Pom) varieties of *P. guajava* L. were air-dried and crushed	UAE: samples were extracted (×3 times) with 15 mL of EtOH: H_2_O mixture (80:20, *v*/*v*) for 10 min at RT, centrifuged for 15 min at 6000 rpm. The pooled supernatants were evaporated, dissolved in 2 mL of MeOH/H_2_O (50:50, *v*/*v*), and stored at −20 °C in the dark until analysis.	HPLC-ESI-Q-TOF-MS	HPLC conditions: Column: Poroshell 120 EC-C18 (100 × 4.6 mm, 2.7 µm); MP: 1% AA in H_2_O (A) and ACN (B) in a gradient mode: 0 min, 0.8% B; 2.5 min, 0.8% B; 5.5 min, 6.8% B; 11 min, 14.4% B; 17 min, 24% B; 22 min, 40% B; 26 min, 100% B; Tª: 25 °C; FR: 0.8 mL/min. ESI conditions: negative ionization mode; capillary: 3500 V; nebulizer pressure: 50 psi; fragment voltage: 3500 V; drying gas FR: 12.0 L/min; gas Tª: 370 °C; N_2_ sheath gas Tª: 350 °C; N_2_ sheath gas FR: 10 L/min; Scan range, *m*/*z:* 50–1500 in full scan mode; collision energy: 30–45 eV.	[GA] (mg/g leaf d.w) (Pyr): 0.060 ± 0.008 (Pom): 0.223 ± 0.003	[73]
Pulp, peel, seed, and husk of Keitt mango	Samples were separated, FD, milled, and kept at −18 °C until use	Solid–liquid extraction: Free polar fraction of mango (FPF): FD powder samples were dissolved in 10 mL of solution of MeOH/H_2_O (80:20, *v*/*v*) and placed for 15 min at RT in an ultrasonic bath. The mixture was centrifuged for 15 min at 1000× *g*, and the supernatant was stored. This process was repeated twice.	HPLC-DAD-ESI-Q-TOF-MS	HPLC conditions: Column: Poroshell 120 EC-C18 (100 × 4.6 mm, 2.7 µm); MP: 1% AA in H_2_O (A) and ACN (B) in a gradient mode: 0 min, 0.8% B; 5.5 min, 6.8% B; 16 min, 20% B; 20 min, 25% B; 25 min, 35% B; 29 min, 100%; Tª: 25 °C; FR: 0.8 mL/min. λ: 240, 280, and 330 nm. ESI conditions: negative ionization mode; capillary: 3500 V; nebulizer pressure: 50 psi; fragment voltage: 3500 V; drying gas FR: 12.0 L/min; gas Tª: 370 °C; N_2_ sheath gas Tª: 370 °C; N2 sheath gas FR: 10 L/min; Scan range, *m*/*z*: 50–1500 in full scan mode; collision energy: 30–45 eV.	[GA] (mg/100 g leaf d.w) FPF Pulp: 2.08 ± 0.02 Peel: 12.18 ± 0.39 Seed: 17.55 ± 0.74 Husk: 2.48 ± 0.05 BPF Pulp: 0.019 ± 0.001 Peel: 0.718 ± 0.062 Seed: 0.310 ± 0.015 Husk: 0.487 ± 0.017	[74]
Red and yellow araçá (*Psidium cattleianum* Sabine) (Pelotas, Brazil)	Red araçá (RA) and yellow araçá (YA) were separated and lyophilized for 72 h. FD samples were milled and stored at −18 °C until analysis	Extraction of extractable phenolic compounds (EPC): 5 mL of MeOH: H_2_O (8:2, *v*/*v*) acidified with 0.35% FA was added to FD samples and vortexed for 3 min. The extract was centrifuged at 3000× *g* for 5 min (4 °C), and the supernatant was placed in a rotary evaporator to remove the MeOH. Extraction of non-extractable phenolic compounds (NEPC): The NEPC fraction was obtained from acid hydrolysis of the solid product generated in the EPC extraction. The pellet was added to 20 mL of MeOH acidified with HCl (15%, *v*/*v*) for 15 min at 90 °C. The extract was centrifuged and placed in a rotary evaporator as before.	LC-DAD-ESI-Q-TOF-MS/MS	HPLC conditions: Column: C18 Synergy Hydro-RP column (250 × 4.6 mm, 4 µm); MP: 0.5% FA in H_2_O (A) and 0.5% FA in ACN (B) in a gradient mode: 99:1 (*v*/*v*) A/B to 50:50 (*v*/*v*) A/B over 50 min and then from 50:50 (*v*/*v*) A/B to 1:99 (*v*/*v*) A/B over 5 min. λ: 280 and 320 nm. ESI conditions: negative ionization mode; capillary: 3000 V; nebulizer pressure: 50 psi; fragment voltage: 3500 V; drying gas FR: 8.0 L/min; gas Tª: 370 °C; N_2_ sheath gas Tª: 310 °C; N2 sheath gas FR: 10 L/min; Scan range, *m*/*z*: 50–1500 in full scan mode; collision energy: 30–45 eV.	[GA] (μg/g) RA-EPC: 9.9 ± 0.5 RA-NEPC: 34.1 ± 2.1 YA-EPC: 7.4 ± 0.3 YA-NEPC: 11.1 ± 0.9	[75]
Seven samples of different types of red wine (Bahia, Brazil)	Samples were stored at 4 °C in the dark: Shiraz (1), Cabernet Sauvignon (2), Cabernet Sauvignon/Shiraz (3), Cabernet Sauvignon/Shiraz (4), Shiraz (5), Shiraz (6), Cabernet Sauvignon/Shiraz (7)	Liquid extraction: NaCl, Na_2_S_2_O_5_, and acidified ethyl acetate were added to each wine, and samples were subjected to ultrasonication for 7 min. After that, the samples were dried and the solid residue was spiked with pyridine, BSTFA, and 1% TMS.	GS-MS	The Tª program was the following: initial temperature of 80 °C, for 1 min, from 80 to 250 °C with a rate of 20 °C/min, 6 °C/min to 300 °C, and finally increased at 20 °C/min to 320 °C, and held for 24 min. ND about GS-MS procedure	[GA] (mg/L) (1): 21.4 ± 1.7 (2): 27.1 ± 4.0 (3): 47.2 ± 5.7 (4): 49.4 ± 6.0 (5): 46.4 ± 6.3 (6): 56.3 ± 5.6 (7): 54.1 ± 3.7	[76]
Divya-Swasari-Vati (DSV) (Haridwar, India)	ND	Powdered DSV were dissolved in 10 mL H_2_O:MeOH (20:80, *v*/*v*) while sonicated for 20 min.	HPTLC	HPTLC plates: 10 × 10 cm plates for fingerprinting, and 20 × 10 cm plates, for quantification on aluminum-backed plates coated with a 0.20 mm layer of silica gel 60 F_254;_ solvent system: two solvent systems, ethyl acetate/toluene/formic acid (10:9:1, *v*/*v*/*v*), and ethyl acetate/formic acid/acetic acid/water (10:1:1:2.3, *v*/*v*/*v*/*v*).	[GA]: 3226.0 ± 610.4 µg/g	[77]
Honey (Western Australia)	Four different varieties of honey were collected: (A) *Calothamnus* spp. honey (B) *Agonis flexuosa* honey (C) *Corymbia calophylla* honey (D) *Eucalyptus marginata* honey	Liquid–liquid extraction: 2 mL of deionized water was added to the honey sample and vortexed. The resulting solution was then extracted three times with 5 mL DCM and ACN (1:1, *v*/*v*). The combined extracts were dried and stored at 4 °C.	HPTLC	HPTLC plates: silica gel 60 F254 plates 10 × 20 cm; solvent system: toluene/ethyl acetate/formic acid (2:8:1, *v*/*v*/*v*) and toluene/ethyl acetate/formic acid (6:5:1, *v*/*v*/*v*); λ: 254 nm.	[GA] (µg/g) (A): 1.64 ± 0.00 (B): n.d. (C): 5.84 ± 0.00 (D): n.d.	[78]
*Dodonaea angustifolia* leaves (DALs) and flower (DAF) (Ethiopia)	Samples were washed, cut into smaller pieces (<45 µm), dried at RT, and ground	UAE: samples were extracted (twice) in 25 mL of MeOH at 35 °C for 15 min. The extracts were centrifuged at 10× *g* for 20 min and the supernatant was filtered and stored.	HPTLC	HPTLC plates: precoated silica gel 60 F254 aluminum plates (20 × 10 cm, 100 µm); solvent system: toluene/ethyl acetate/FA/MeOH (20:12:4:8, volume ratio); λ: 254 to 450 nm; humidity: 44–46%	[GA] (mg/100 g) DAL: 32.26 ± 1.55 DAF: 53.64 ± 1.21	[79]
Leaves of *Ricinus communis* Linn (Shanghai, China)	Three samples of the leaves of *R. communis* L (1, 2, 3) were all dried at 60 °C for 2 h and then pulverized.	Powdered samples were dispersed in MeOH in a water bath at 60 °C for 3 h. After cooling, it was sonicated for 30 min and filtered. The extract was diluted using 50 mM borate buffer (pH 9.0) before CE analysis.	CE-AD	A ±30 kV high-voltage dc power supply provided a separation voltage between the ends of the capillary. The inlet of the capillary was held at a positive potential and the outlet of the capillary was maintained at ground. The separations were carried out in a 40 cm length of 25 µm i.d. and 360 µm o.d. fused silica capillary. The CE system was assembled at Tª 25 °C. The detection electrode was a 300 µm diameter carbon disc electrode at a detection potential of +0.90 V and a saturated calomel electrode (SCE), as the reference electrode was used in combination with a BAS LC-4C amperometric detector.	[GA] (mg/g) (1): 11.48 (2): 9.627 (3): 6.778	[80]
Alperujo samples from one olive oil (Córdoba, Spain)	Samples were taken directly from the production line and stored at −20 °C until analysis	Alperujo was placed in MeOH–H_2_O (1:3, *v*/*v*) for 13 min under ultrasonic irradiation (duty cycle 0.5 s, output amplitude 10%, applied power of 450 W, with the probe placed at 3 cm from the top surface of the extraction cell). During extraction, FR changed at 2 mL/min every 40 s, and after extraction was completed, DMF was added to the extract. Then, the sample was diluted using MeOH, and before introduction into the CE system, the extract was centrifuged for 3 min at 3000 rpm.	CE-DAD	The running buffer used was a solution of 45 mM H_3_BO_3_ (pH 9.6), adjusted with NaOH to pH 10 and with 5% MeOH. Extracts were electrokinetically injected by application of 25 kV for 4 s. The analysis voltage was 27 kV, being the average current ∼110 A, Tª 30 °C, and λ: 210 nm. To maintain the capillary under optimal working conditions, its surface was regenerated after each run by sequential washing with water (2 min), 0.1 M sodium hydroxide (2 min), 1 min waiting, followed by the running buffer (10 min).	[GA]: 12.48 ± 0.4 µg/g	[81]
Leaves from rosemary (*Rosmarinus officinalis*), sage (*Salvia officinalis*), oregano (*Origanum vulgare*), and *Ligustrum lucidum* (Ioannina, Greece)	The samples were washed and pulverized into a fine powder	Oregano leaves were extracted with acetone in a Soxhlet apparatus for 6 h, while sage, rosemary, and *L. lucidum* leaves were subsequently extracted with hexane and ethyl acetate in a Soxhlet apparatus for 6 h. Furthermore, *L. lucidum* leaves were subjected to two more treatments: macerated in MeOH for 7 days in the dark at RT and boiled with distilled water for 1 h. All the extracts were filtered, freeze-dried, and stored at −20 °C.	^1^H-NMR	NMR experiments were performed at 295 K on a Bruker 500 spectrometer equipped with a TXI cryoprobe. Samples were dissolved in DMSO-*d*_6_. All chemical shifts were measured with reference to the internal standard TSP-*d*_4_ (δ = 0.000 ppm) of a given concentration (0.03 mM). All spectra were acquired with an acquisition time of 1.818 s, relaxation delay 5 s, 64 K data points, 90° pulse length, and optimum low-power radiofrequency irradiation for the water signal pre-saturation.	^1^H NMR spectrum of the artificial mixture of phenolic acids found 2.94 mM GA	[82]
Green tea samples (*C. sinensis*) (Guangzhou, China)	Different varieties of green tea were pulverized and stored until use.	1.5 mL Milli-Q water was added to 50 mg pulverized green tea and kept at 70 °C with continuous shaking for 25 min. The extract was centrifuged at 13,000 rpm for 20 min, and the supernatant was stored until analysis.	^1^H-NMR	600 µL of the sample was mixed with 100 µL of TSP-d_4_–D_2_O solution and analyzed in a Bruker 600 spectrometer at 600.13 MHz proton frequency; 128 scans of 38.460 data points were acquired, with a spectral width of 9600 Hz (16 ppm), pulse width of 12.34 ms, acquisition time of 4.0 s, relaxation D1 of 10 s, flip angle of 90°, and constant gain of 181.	[GA] (mg/g) (1) 0.34 (2) 1.58 (3) 1.88 (4) 1.31 (5) 1.73 (6) 1.63	[83]
Leaves from green tea of the cultivars *C. sinensis Yabukita* and *Yutakamidori*	ND	100 mg of green tea from each cultivar was sprayed with liquid N_2_ and subjected to microextraction in 1 mL of CD_3_OD (0.05% TMS). Then, sonication and centrifugation were performed at 12,000 rpm for 10 min Each, and the supernatant was stored until analysis.	^1^H HR-MAS NMR	^1^H HR-MAS qNMR spectra were acquired on a Bruker 400 (9.4 T) spectrometer at 400.13 MHz, equipped with 4 mm four channel (^1^H; ^13^C; ^15^N; ^2^H) HR-MAS probe and gradient field in the direction of the magic angle (θ = 54.74°). All acquisitions were performed with an interpulse delay time of 5 × T_1_, a recycle delay of 20 s (D1), 256 transients, AQ of 4.89 s, and 64 K points during the acquisition, using a spectral window of 8012.820 Hz.	GA was identified via a singlet at δ_H_ 7.10	[84]
Different water samples matrices, including tap (1), mineral (2), and river (3) (Iran)	Before the determination of GA in samples, 500 mL of each sample were filtered through Whatman filter paper, and the samples were stored at 4 °C until analysis and processed within 1 week of collection	An ultrasonic processor operated at 40 kHz with a power of 130 W was used as the source of ultrasound for the enhanced recovery of GA.	UV–Vis	The volume of eluent (EtOH), sonication time, amount of sorbent, and pH were the parameters to optimize.	[GA] (ng/mL) (1): 995.43 (2): 984.35 (3): 990.71	[85]
Green lentils (Saskatoon, SK, Canada)	Initial preparation of the samples involved the removal of lipids: samples were dried, dehulled. and defatted with hexane (1:5, *w*/*v*) for 5 min at RT. This procedure was repeated twice more, and samples were stored at −20 °C. Black hull soluble (BHS); black whole soluble (BWS); black dehull soluble (BDS); green hull soluble (GHS); green whole soluble (GWS); green dehull soluble (GDS); black hull insoluble-bound (BHI); black whole insoluble-bound (BWI); black dehull insoluble-bound (BDI); green hull insoluble-bound (GHI); green whole insoluble-bound (GWI); green dehull insoluble-bound (GDI)	Soluble phenolic compounds (SPCs): 10 mL of MeOH/acetone/H_2_O (1:1:1, *v*/*v*/*v*) was added to defatted samples and then sonicated for 20 min at 40 °C. The supernatant was then filtered and stored. Non-soluble phenolic compounds (NSPCs): Residues after the extraction of soluble phenolics were hydrolyzed using 2 M NaOH while stirring for 4 h at RT, then using 6 M HCl, and then centrifuged at 2000× *g* for 5 min. The supernatant was then extracted with hexane and then with diethyl ether/ethyl acetate 1:1 (*v*/*v*), four times. The solvent was then removed using a rotary evaporator, and then reconstituted in MeOH and stored at −20 °C until use.	(ESI)-MS-MS	500 µL of both SPC and NSPC were injected into a mass spectrometer through direct diffusion at a rate of 10 µL/min. The individual phenolic compounds were identified and quantified in the negative mode along with 4045 (v) ion spray voltage, 16.1 (arb) sheath gas, 2.4 aux gas, 325 °F ion transfer tube Tª, and 30 °C vaporizer Tª.	[GA] (μg/g) SPC: (BHS): 96.0 ± 2.0 (BWS): 2.8 ± 0.5 (BDS): 0.9 ± 0.2 (GHS): 4.0 ± 0.2 (GHS): 1.4 ± 0.1 (GDS): 1.1 ± 0.0 NSPC: (BHI): 489.9 ± 38.9 (BWI): 1.6 ± 0.5 (BDI): 1.5 ± 0.2 (GHI): 104.1 ± 2.4 (GHI): 0.9 ± 0.0 (GDI): 2.6 ± 0.2	[86]
Alcohol beverages (sherry and fruit wines and cognac 1 and 2)	No sample pretreatment is necessary and the procedure is rapid	A test solution containing 5–150 µg of GA, 1.5 mL of fresh 8 × 10^−3^ M 4-nitrobenzenediazonium tetrafluoroborate, 2.5 mL of 1 M HCl, and water up to a volume of 25 mL was sequentially added to vessels with ground stoppers. A single (polyurethane foam) PUF tablet was placed in each vessel, and after an unspecified period, tablets were removed and dried, and their diffuse reflectance was measured.	DRS	Diffuse reflectance values were measured on “Uniphot” portable reflectometer–colorimeter. The GA concentration was obtained using a Kubelka–Munk calibration curve, *F* (*R*) = *f* (*C*), where F (R) is the Kubelka–Munk function at 410 nm and C is the concentration of GA in µg/mL	[GA] (µg/mL) Sherry wine: 43 ± 3 Fruit wine: 77 ± 5 Cognac 1: 18 ± 2 Cognac 2: 38 ± 2	[87]

Abbreviations: Mobile phase (MP); temperature (Tª); freeze-dried (FD); ethanol (EtOH); methanol (MeOH); water (H_2_O); Acetonitrile (ACN); acetic acid (AA); phosphoric acid (PA); hydrochloric acid (HCl); Tetrahydrofuran (THF); trifluoroacetic acid (TFA); Dichloromethane (DCM); room temperature (RT); flow rate (FR); detection (λ); no data (ND); relative humidity (RH); Reversed-Phase High-Performance Liquid Chromatography with Diode Array Detection (RP-HPLC-DAD); High-Performance Thin-Layer Chromatography (HPTLC); High-Performance Liquid Chromatography coupled with Electrospray Ionization Quadrupole Time-of-Flight Mass Spectrometry (HPLC-ESI-Q-TOF-MS); triple quadrupole (QqQ); UAE: ultrasonic-assisted extraction (UAE); not detected (n.d.); High-Resolution Magic-Angle Spinning Nuclear Magnetic Resonance Spectroscopy (HR-MAS NMR); Tetramethylsilane (TMS); delay time (D1); acquisition time (AQ); diffuse reflectance spectrometry (DRS); dimethyl sulfoxide (DMSO); sodium chloride (NaCl); sodium metabisulfite (Na_2_S_2_O_5_); N,O-Bis(trimethylsilyl)trifluoroacetamide (BSTFA); trimethylsilyl (TMS); ultraviolet–visible spectrophotometry (UV–Vis); Capillary Electrophoresis (CE) with Amperometric Detection (AD); N,N-dimethylformamide (DMF).

**Table 3:** Recent studies on in vivo antioxidant activity of GA.

Study Model and Administration Way	Dose mg/kg	GA Antioxidant Activity	Therapeutic Outcome	References
Mice with diabetic nephropathy induced with methylglyoxal, oral administration	30	Decreases MDA, miR-192, miR-204, albuminuria, and Nrf2; increases antioxidant enzymes such as SOD, CAT, glyoxalase1, and miR-29a	Mitigates kidney damage by reducing oxidative stress markers	[144]
Elastase emphysema in rats, oral administration	30	Lowers MDA and NF-κB levels; increases GS, SOD, CAT, Nrf2, and HO-1 levels	Reduces ischemia/reperfusion injury and histological damage	[145]
Paraquat liver injury in rats, oral administration	50 or 100	Lowers TG, AST, ALT, ALP, MDA, PC, IL-1β, LDL-C, and VLDL-C levels; increases HDL-C, FRAP, SOD, and CAT levels	Improves histological damage	[150]
Metabolic syndrome in rats, oral administration	20	Increases SOD, CAT, GPx, and GSH; decreases ROS, LPO, TNF-α, and IL-1β	Reduces oxidative stress and enhances recognition memory, hippocampal dendritic spines, and antioxidant enzymes	[161]
Paclitaxel neuropathy in mice, intravenous injection	20 or 40	Lowers LPO, TNF-α, and MPO levels; increases GSH level	Reduces thermal and mechanical hyperalgesia and allodynia symptoms	[152]
Quinolinic acid-induced neurotoxicity in rats, oral administration	200	Increases GPx and CAT levels; decreases caspase-3, IL-1β, IL-6, and TNF-α levels	Protects against oxidative damage	[153]
Carbon tetrachloride liver fibrosis in rats, oral administration	100	Lowers AST, ALT, ALP, bilirubin, albumin, and MDA; increases SOD, CAT, and GSH levels	Prevents oxidative damage in emphysema	[154]
Streptozotocin diabetes in rats, oral administration	10, 50, 100	Decreases LPO and increases GSH levels	Lowers oxidative stress and improves depressive-like behavior	[156]
COPD-linked lung inflammation/emphysema in mice, intraperitoneal injection	100	Lowers IL-6, TNF-α, IL-1β, ROS, LPO, PC, MMP-9, MMP-2; increases GSH and TIMP-1 levels	Decreases oxidative stress and histological damage	[162]
Nicotine-induced testicular injury in mice, intraperitoneal injection	20	Lowers NO and MDA levels; increases FRAP and SOD levels	Reduces histological damage and increases sperm quality and testosterone	[163]
Sodium arsenite-induced toxicity in rats, oral administration	30	Lowers creatine kinase-MB, MDA, and NO levels; increases white blood cells, platelets, MCV, hemoglobin, MCH, GPx, GSH and SOD levels	Reduces histological damage of the heart	[159]
Isoproterenol-induced ischemia/reperfusion in rats, oral administration	50	Increases SERCA2a and SOD levels; lowers LDH and creatine kinase-MB levels	Improves cardiac function and reduces cardiac hypertrophy	[160]
STZ diabetes in rats, oral administration	20 or 40	Reduces MDA markers; enhances antioxidant enzymes like GSH, SOD, and CAT	Reduces oxidative stress and prevents glomerular damage and tubulo-interstitial fibrosis	[164]
Bisphenol A toxicity in rats, oral administration	50 or 200	Scavenges ROS; boosts SOD, CAT, and GSH antioxidants; decreases levels of urea, LPO, creatinine, uric acid, IL-6, and IL-1β	Mitigates oxidative damage and reduces kidney morphological damage	[165]
Paraquat toxicity in rats, oral administration	50 or 100	Lowers uric acid, creatinine, PCI, IL-1β, and MDA levels; enhances SOD and CAT activity	Reduces oxidative stress and kidney morphological damage	[166]
Diclofenac toxicity in rats, oral administration	50 or 100	Reduces serum urea, uric acid, creatinine, MDA, IL-1β, and NO levels; enhances SOD, CAT, GPx, and GSH levels	Prevents oxidative damage and kidney morphological damage	[167]
Cyclophosphamide toxicity in mice, oral administration	30	Reduces BUN, KIM-1, NGAL, IL-1β, TNF-α, and creatinine levels; enhances antioxidants like SOD, CAT, GPx, and GSH	Mitigates kidney damage by reducing oxidative stress markers	[168]
Cisplatin toxicity in rats, oral administration	40	Lowers creatinine, LncRNA TUG1, Bax, caspase-3, Bcl-2, BUN, MDA, IL-1β, and TNF-α levels	Prevents oxidative damage and kidney morphological damage	[169]
Glyoxylic acid kidney stone formation in mice, intraperitoneal injection	50	Decreases MDA, Lcn2 mRNA, KIM1 mRNA, creatinine, BUN, OPN, TNF-α, IL-1β, renal tubular injury, and CaOx crystal deposition; increases 4-HNE, Nrf2, HO-1 levels	Reduces deposition of kidney stones and oxidative stress	[170]
Hyperuricemia in mice, oral administration	100	Decreases BUN, uric acid, Cys-C, MDA, IL-1β, COX-2, TGF-β1, and GLUT9 levels; increases CAT, OAT1, OAT3, and GPx levels	Reduces kidney morphological damage	[171]
Hypoxic-ischemic brain damage in rats, intraperitoneal injection	50	Decreases IL-1β, ROS, and LPO levels; increases SOD and CAT levels	Reduces neuronal loss, motor ability issues, and oxidative stress markers, and learning and memory improved	[40]
Hypothyroidism in rats, oral administration	50	Lowers ATF4, PERK1, p-eIF2α, GADD153, GADD34, caspase-12, Bax, ATF6α, IRE1a, and p53; increases eIF2α and Bcl-2 levels	Prevents oxidative damage and reduces morphological damage in CA3 hippocampal region	[172]
Ketamine toxicity in rats, oral administration	10, 25, 50	Lowers ROS, PC, and LPO levels; increases NPSH levels	Reduces oxidative stress	[173]
HFD obesity in db/db mice, oral administration	100	Lowers AST, ALT, cholesterol, TG, LPO, SREBP1, and SREBP2 levels; increases GST, GPx, SOD, and CAT levels	Reduces oxidative stress in pulmonary fibrosis and histological damage	[174]
Cyclophosphamide toxicity in mice, oral administration	100, 200, or 400	Increases SOD and GSH levels	Improves histological damage, reduces micronucleus and DNA strand breaks, and decreases oxidative stress markers by increasing antioxidant enzymes	[175]
Thioacetamide liver fibrosis in rats, oral administration	20	Lowers AST, total bilirubin, ALT, ALP, MDA, TGF-β1, p-Smad3, and miR-21 levels; increases miR-30, SOD, CAT, and miR-200 levels	Improves histological damage	[176]
Zearalenone reproductive dysfunction in rats, oral administration	40	Increases CAT, GPx, GST, GSH, SOD and TSH levels; decreases RONS, LPO, MPO, NO, and TNF-α levels	Increases testicular function enzymes, reproductive hormones, and sperm quality; reduces histological damage	[177]
Cisplatin ovarian damage in rats, oral administration	2.5 or 5	Lowers MDA, TNF-α, and caspase-3 levels; increases CAT level	Reduces histological damage	[178]
TAC cardiac hypertrophic remodeling in mice, oral administration	20	Lowers IL-1β, IL-6, gp130, CaNA, p-ERK1/2, MCP-1, EGFR, p-STAT3, and p-AKT levels	Reduces myocardial dysfunction, cardiac hypertrophy, and histological damage	[179]

Alphabetical list of abbreviations used in Table 3: 4-HNE (4-Hydroxynonenal), ALP (Alkaline Phosphatase), ALT (Alanine Aminotransferase), AST (Aspartate Aminotransferase), ATF4 (Activating Transcription Factor 4), ATF6α (Activating Transcription Factor 6 alpha), Bax (Bcl-2-associated X protein), Bcl-2 (B-cell lymphoma 2), BUN (Blood Urea Nitrogen), CaNA (Calcineurin A), CaOx (Calcium Oxalate), Caspase-12 (Cysteine-aspartic protease 12), CAT (catalase), COPD (Chronic Obstructive Pulmonary Disease), COX-2 (Cyclooxygenase-2), Cys-C (Cystatin-C), EGFR (Epidermal Growth Factor Receptor), FRAP (Ferric Reducing Antioxidant Power), GADD153 (Growth Arrest and DNA Damage-inducible protein 153), GADD34 (Growth Arrest and DNA Damage-inducible protein 34), GLUT9 (Glucose Transporter 9), GPx (Glutathione Peroxidase), GS (Glutamine Synthetase), GSH (glutathione), GST (Glutathione S-Transferase), HDL-C (High-Density Lipoprotein Cholesterol), HO-1 (Heme Oxygenase-1), IL (interleukin) with specific mentions of IL-6 and IL-1β, IRE1α (Inositol-Requiring Enzyme 1 alpha), KIM-1 (Kidney Injury Molecule-1), LDH (Lactate Dehydrogenase), LDL-C (Low-Density Lipoprotein Cholesterol), LncRNA (Long non-coding RNA), LPO (lipid peroxidation), MCH (Mean Corpuscular Hemoglobin), MCV (Mean Corpuscular Volume), MCP-1 (Monocyte Chemoattractant Protein-1), MDA (malondialdehyde), miR (MicroRNA) including miR-192, miR-204, miR-21, miR-29a, and miR-30, MMP-2 (Matrix Metalloproteinase 2), MMP-9 (Matrix Metalloproteinase 9), MPO (Myeloperoxidase), NF-κB (Nuclear Factor kappa-light-chain-enhancer of activated B cells), NGAL (Neutrophil Gelatinase-Associated Lipocalin), NO (nitric oxide), NPSH (Non-Protein Sulfhydryl), Nrf2 (Nuclear factor erythroid 2-related factor 2), OAT1 (Organic Anion Transporter 1), OAT3 (Organic Anion Transporter 3), OPN (Osteopontin), p-AKT (Phosphorylated Protein Kinase B (AKT)), p-eIF2α (Phosphorylated eukaryotic Initiation Factor 2 alpha), PCI (Plasma Creatinine Index), PC (protein carbonyl), p-ERK1/2 (Phosphorylated Extracellular Signal-Regulated Kinases 1/2), PERK (Protein kinase RNA-like Endoplasmic Reticulum Kinase), p-Smad3 (Phosphorylated Mothers Against Decapentaplegic Homolog 3), p-STAT3 (Phosphorylated Signal Transducer and Activator of Transcription 3), ROS (reactive oxygen species), RONS (Reactive Oxygen and Nitrogen Species), SERCA2a (Sarco/Endoplasmic Reticulum Ca^2+^-ATPase 2a), SOD (superoxide dismutase), SREBP (Sterol Regulatory Element-Binding Protein) including SREBP1 and SREBP2, STZ (Streptozotocin), TAC (Transverse Aortic Constriction), TG (triglycerides), TGF-β1 (Transforming Growth Factor beta 1), TIMP-1 (Tissue Inhibitor of Metalloproteinases 1), TNF-α (tumor necrosis factor alpha), TSH (Thyroid-Stimulating Hormone), TUG1 (Taurine Upregulated Gene 1), and VLDL-C (Very Low-Density Lipoprotein Cholesterol).

**Table 4:** GA anticancer activity proven in mice and human treatments.

Study Model	Dose	Anticancer Activity	Therapeutic Outcome	References
Glioblastoma cell in rats	50 and 100 mg/kg	Activates mitochondrial apoptotic pathway	90% reduction in tumor size and lower brain oxidative damage	[187]
DU145 prostate cancer cells in mice	25, 50, and 100 µg/mL	Initiates mitochondria-mediated apoptosis, inhibits cell growth at G2/M phases by activating Chk1 and Chk2 and inhibiting Cdc25C and Cdc2 activities	Reduced cancer cell survival and induced cell cycle arrest	[188]
A375S2 human melanoma cells in mice	100 µmol/L	Promotes apoptosis, downregulates Bcl-2, upregulates Bax	Marked inhibition of cell proliferation and induction of apoptosis	[190]
Breast cancer MCF-7 human cell line	GA, paclitaxel, and carboplatin in various concentrations	Cell cycle arrest at the G2/M phase	GA boosted the efficacy of paclitaxel and carboplatin, induced apoptotic cell death in MCF-7 cells, and increased the expression of P53, Bax, and CASP-3	[198]
Non-small-cell lung cancer (NSCLC) in human cells and rats	10, 20, and 40 µg/kg	Inhibits EGFR activation and reduces the CARM1-PELP1 complex	Inhibits proliferation and promotes apoptosis in NSCLC human cells, contributing to reduced tumor growth in vivo in rats	[209]
Colon cancer in rats	20 and 50 mg/kg body weight	Reduces lipid peroxidation products like TBARS, LOOH, and CD, increases antioxidant levels like SOD, CAT, GSH, GR, and GPx, and lowers ascorbic acid and α-tocopherol levels in DMH-treated rats	Strong chemopreventive effect on DMH-induced colon carcinogenesis	[210]
Human bladder cancer T24 cell line	6.25, 12.5, 25 µg/mL	Inhibits cell proliferation by disrupting PI3K/Akt/NF-κB signaling pathways	Inhibits T24 cell proliferation and metastasis, and leads to apoptosis; pro-apoptotic activity linked with mitochondrial dysfunction and PI3K/Akt/NF-κB signaling inhibition	[211]
Leukemia and its resistant sublines (HL60 cell HL60/VINC HL60/M2) in human cells	Eleven concentrations ranging from 10–500 µM	Alters cell cycle distribution and increases cell population in sub-G1 phase, modulates ROS production in a time- and dose-dependent manner	Cytotoxic activity against human promyelocytic leukemia-sensitive HL60 line and its resistant sublines, showing different MDR phenotypes: HL60/VINC (overexpressing P-gp) and HL60/M2	[212]

Alphabetical list of abbreviations used in Table 4: A375S2 cells (human melanoma), Akt (Protein Kinase B), CARM1-PELP1 complex (Coactivator-associated arginine methyltransferase 1 with Proline, Glutamic Acid, and Leucine Rich Protein 1), CAT (catalase), Cdc2 (Cyclin-dependent Kinase 2), Cdc25C (Cell Division Cycle 25C), CD (Conjugated Dienes), Chk1 (Checkpoint Kinase 1), Chk2 (Checkpoint Kinase 2), DMH (Dimethylhydrazine), DU145 cells (prostate cancer), EGFR (Epidermal Growth Factor Receptor), GBM (glioblastoma), GPx (Glutathione Peroxidase), GR (Glutathione Reductase), GSH (glutathione), HL60 cells (promyelocytic leukemia), LOOH (Lipid Hydroperoxides), MCF-7 cells (breast cancer), MDR (multidrug resistance), MX2 (Mitoxantrone), NF-kB (Nuclear Factor kappa-light-chain-enhancer of activated B cells), NSCLC (non-small-cell lung cancer), PI3K (Phosphoinositide 3-kinases), ROS (reactive oxygen species), SOD (superoxide dismutase), T24 cells (bladder cancer), TBARS (Thiobarbituric Acid Reactive Substances), and VINC (Vincristine).
